# FlySilico: Flux balance modeling of *Drosophila* larval growth and resource allocation

**DOI:** 10.1038/s41598-019-53532-4

**Published:** 2019-11-20

**Authors:** Jürgen Wilhelm Schönborn, Lisa Jehrke, Tabea Mettler-Altmann, Mathias Beller

**Affiliations:** 1 Institute for Mathematical Modeling of Biological Systems, Heinrich Heine University, Duesseldorf, Germany; 2 Systems Biology of Lipid Metabolism, Heinrich Heine University, Duesseldorf, Germany; 3 Institute of Plant Biochemistry & Cluster of Excellence on Plant Sciences, Heinrich Heine University, Duesseldorf, Germany

**Keywords:** Biochemical reaction networks, Metabolism, Drosophila

## Abstract

Organisms depend on a highly connected and regulated network of biochemical reactions fueling life sustaining and growth promoting functions. While details of this metabolic network are well established, knowledge of the superordinate regulatory design principles is limited. Here, we investigated by iterative wet lab and modeling experiments the resource allocation process during the larval development of *Drosophila melanogaster*. We chose this system, as survival of the animals depends on the successful allocation of their available resources to the conflicting processes of growth and storage metabolite deposition. First, we generated “FlySilico”, a curated metabolic network of *Drosophila*, and performed time-resolved growth and metabolite measurements with larvae raised on a holidic diet. Subsequently, we performed flux balance analysis simulations and tested the predictive power of our model by simulating the impact of diet alterations on growth and metabolism. Our predictions correctly identified the essential amino acids as growth limiting factor, and metabolic flux differences in agreement with our experimental data. Thus, we present a framework to study important questions of resource allocation in a multicellular organism including process priorization and optimality principles.

## Introduction

Balancing limited resources to concurrent processes is an essential task in all areas of life. Every organism, for example, needs to allocate its available resources – mostly in the form of diet-derived nutrients – to concurrent processes such as live sustaining functions, reproduction, or storage metabolite synthesis. While the metabolic pathways involved in these processes, as well as some regulatory signaling pathways, are well established, the overarching design principles governing resource allocation and prioritization are elusive^1,2^. This is especially true for multicellular heterotrophic organisms, which often have an almost unlimited amount of destinations for channeling their available resources. On top of a plethora of energy consuming processes available to the whole organism (physical movement, growth and reproduction, energy storage metabolite deposition), higher order multicellular organisms are composed of a multitude of organs. This results in an even higher complexity, given that many organs have distinct and different metabolic preferences, which is important during health and disease states^3–5^. The mammalian brain, for example, depends on sugars for energy production, whereas most other body cells can additionally utilize other energy liberating pathways such as fatty acid beta-oxidation. To this end, a bottom-up understanding of the resource allocation regulation therefore appears close-to impossible based on this complexity and lack of detailed information. Thus, an abstraction in the form of a top-down modeling paradigm has the potential to reveal design principles, which serve as a starting point to investigate resource allocation principles. Yet, the various degrees of freedom available to complex organisms complicate the modeling procedures, as model solving usually targets the optimization (maximizing or minimizing) of a distinct objective function such as e.g. biomass production or growth^6^. Given that multicellular organisms can have multiple and often conflicting objective functions (e.g. reproduction, longevity), or objective functions without the aim of maximization or minimization (e.g. metabolic processes to sustain survival, growth in terms of sustaining healthy cell turnover, deposition of energy depots), the identification of a single and clear-cut objective function is difficult.

**Figure 1:**
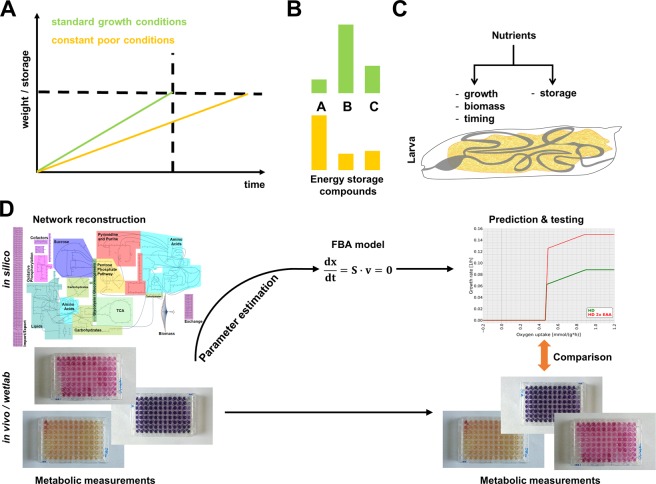
Plasticity of resource allocation and experimental design of the study. (**A**) Development involves growth and weight gain (in part due to the deposition of storage metabolites) over time. Altered environmental conditions, such as a rich (green) or poor (orange) nutrition results in an altered timing and/or (**B**) altered energy storage compound levels (compounds **A**–**C** show a different relative abundance under rich (green) and poor (orange) nutritional conditions). (**C**) Nutrients fuel conflicting processes. *Drosophila* larvae need to channel available nutrients to sustain either growth or the formation of storage metabolites. Following nutrient uptake in the gut (shown in grey) copious amounts of triglyceride and glycogen stores are built in the fat body (yellow), which largely fills the body cavity of developing larvae. (**D**) Our experimental design involved *in silico* and *in vivo* (wet lab) experiments. For the *in silico* studies, we first reconstructed main parts of the *Drosophila* metabolic network. For the parameter estimation, we performed metabolic profiling experiments during the *Drosophila* larval development. Subsequently, the metabolic network served the constraint-based flux balance modeling (FBA). With the FBA models, we predicted the consequences of alterations of the dietary composition and validated the modeling results using targeted experiments. High resolution versions of the metabolic network shown in (**D**) are provided in Fig. 2 as well as an interactive version of the metabolic network as supplemental data.

Development, however, appears to represent an exception. The development of most organisms follows a stereotyped program, which involves hard constraints e.g. in terms of the timing or metabolic thresholds. Holometabolous insects, for instance, need to deposit sufficient energy storage amounts to allow metamorphosis into an adult organism, which is often associated with a minimal weight, which is also termed critical weight in insects such as *Drosophila melanogaster*^7^. Given the variations many organisms face during development – based on e.g. fluctuation in temperature or food supply and quality – developmental programs are at the same time also flexible (Fig. 1A,B). The time needed to reach a certain weight or storage threshold necessary to complete development thus can be extended under poor nutritional conditions, for example (Fig. 1A). Besides adjusting the developmental timing, organisms can also alter the relative amounts of their energy storage compounds (Fig. 1B). Thus, development of multicellular organisms should be accessible to modeling campaigns, as there is a clear-cut objective function (growth) and sufficient plasticity (timing and metabolic fine-tuning) to test modeling predictions. A better knowledge of the resource allocation principles potentially answers the question whether the developmental growth and resource allocation are in multicellular organisms also (pareto-)optimal. A pareto-optimality was previously reported for bacterial growth and metabolism^8^. Pareto-optimality was also proposed for certain phenotypic traits of multicellular organisms^9,10^, yet this is still under discussion^11,12^.

Here, we use the larval development of *Drosophila melanogaster* as a model system for the analysis of the resource allocation of a multicellular organism using *in silico* and wet lab experiments (Fig. 1C,D). This system is particularly well suited for this endeavor based on the following key-points: (i) Larval development of *Drosophila* involves a massive increase in size and weight coupled to the deposition of copious energy stores necessary to fuel metamorphosis (Fig. 1C)^13^. In order to allow this massive size increase, larvae are constantly eating^14^, which facilitates the estimation of energy expenditure and metabolite intake. (ii) *Drosophila* larval development and resource allocation shows an inherent plasticity. Poor nutritional conditions, for example, result in a prolonged developmental timing based on a lowered rate of development (Fig. 1A)^15^. This change of developmental timing is often paralleled by an altered metabolite composition of the organism^16–19^. Intriguingly, also e.g. mammals adapt their metabolism to *in utero* nutritional alterations^20^ and the impact of the nutritional status during development and early life on the later stages is well described in terms of the life history theory^21,22^ and the process of metabolic programming^23–25^. (iii) For the modeling procedures, we benefitted from a previously published chemically defined, fully synthetic minimal *Drosophila* food (*Holidic diet*; HD)^26^, which allowed us to clearly define the input of our model. On top of the general advantages of working with small animals, such as the large number of progeny and accessibility of the developmental stages, these characteristics clearly facilitated our investigations.

To target resource allocation in *Drosophila* larval development, we generated the – to the best of our knowledge – to date largest curated metabolic network for fruit flies and subjected it to flux balance analyses. To validate and optimize our metabolic network, we used targeted metabolite quantifications and GC-coupled mass spectrometry metabolomics measurements of larvae grown on the HD. We built the model capturing previously known requirements of *Drosophila* metabolism, such as sterol auxotrophy^27^, and tailored it to incorporate all prominent ingredients of the HD. Our model predictions allowed us to correctly identify the amount of essential amino acids as growth limiting factor. Further, the model predictions resulted in flux differences, which are in line with the measured metabolite alterations associated with growth of the larvae in food with elevated amounts of sucrose or essential amino acids. These proof-of-principle experiments provide a starting point to investigate the optimality principles of multicellular resource allocation.

## Results

### Drosophila metabolic network reconstruction

**Figure 2:**
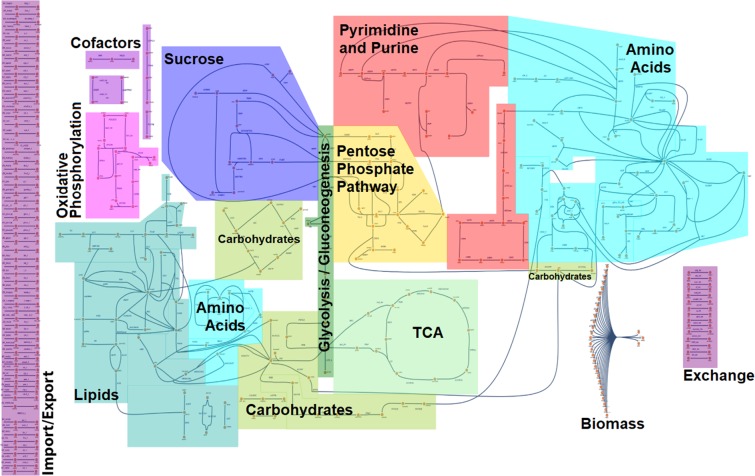
Map of the FlySilico V1.0 metabolic model of *Drosophila melanogaster*. On top of the central carbon metabolism, we included reaction complexes such as the lipid or amino acid metabolic pathways. A main goal was to include the pathways necessary for metabolizing the main constituents of the holidic diet and the pathways covering our experimentally quantified metabolites. For details, please see main text, Table S1 and Interactive Supplementary Fig. 1.

In order to model *Drosophila* larval growth and resource allocation, we first constructed a flux balance capable metabolic network covering the biochemical pathways necessary to metabolize the major constituents of the minimal, synthetic medium (*Holidic diet*; HD)^26^, which we used to grow the fruit flies during the wet lab procedures. On top of the central carbon metabolism, we therefore included e.g. amino acid, lipid, and carbohydrate metabolism (Fig. 2, Table S1, and Interactive Supplementary Fig. 1). In total, our model – termed FlySilico – covers 363 reactions and 293 metabolites. To date, there are surprisingly only two other *Drosophila* metabolic networks available. The first one focuses on the effects of hypoxia on ATP production^28–30^. The other one is a whole-genome computer generated model, which lacks curation (BMID000000141998; https://www.ebi.ac.uk/biomodels-main/BMID000000141998).

**Figure 3:**
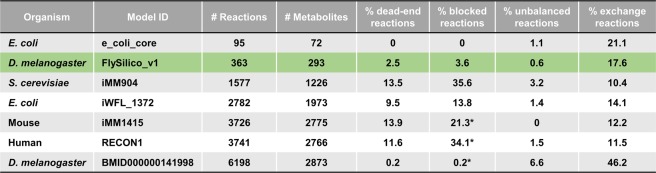
Comparison of FlySilico V1.0 to other publically available metabolic networks. Dead-end reactions result in the production of metabolites, which are not further utilized in the network. Blocked-reactions are not accessible during the solving of the network. Unbalanced reactions are violating the conservation of mass and/or charge. Exchange reactions represent either biologically necessary transporters (such as for the import of nutrients into the system, or naturally occurring transporters for the export of end products), or transport reactions necessary for the modeling, to e.g. eliminate metabolites which are not further processed as the necessary biochemical reactions were omitted from the model. The values marked by an asterisk were calculated using non-loopless conditions. All other values are determined using loopless computations.

For our model reconstruction, we started from scratch and emphasized on avoiding biologically unfeasible reactions (dead-end, blocked, and unbalanced reactions) as well as on minimizing the number of exchange reactions (see methods section). Figure 3 shows a comparison between different aspects of our FlySilico and a selection of previously published other FBA-models of different organisms including the whole genome *Drosophila* model. While of course still limited in size, our model has a low amount of biologically unfeasible reactions. The importance of this became evident when we performed simulations with the whole genome, *in silico* generated *Drosophila* metabolic network. Here, simulations resulted in a positive Biomass production (Fig. S1) even without any inputs entering the model; i.e. that this model allows perpetual motion. For FlySilico, we did not detect such an artificial and erroneous behavior (data not shown).

### Development of a Drosophila biomass function based on experimental data

**Figure 4:**
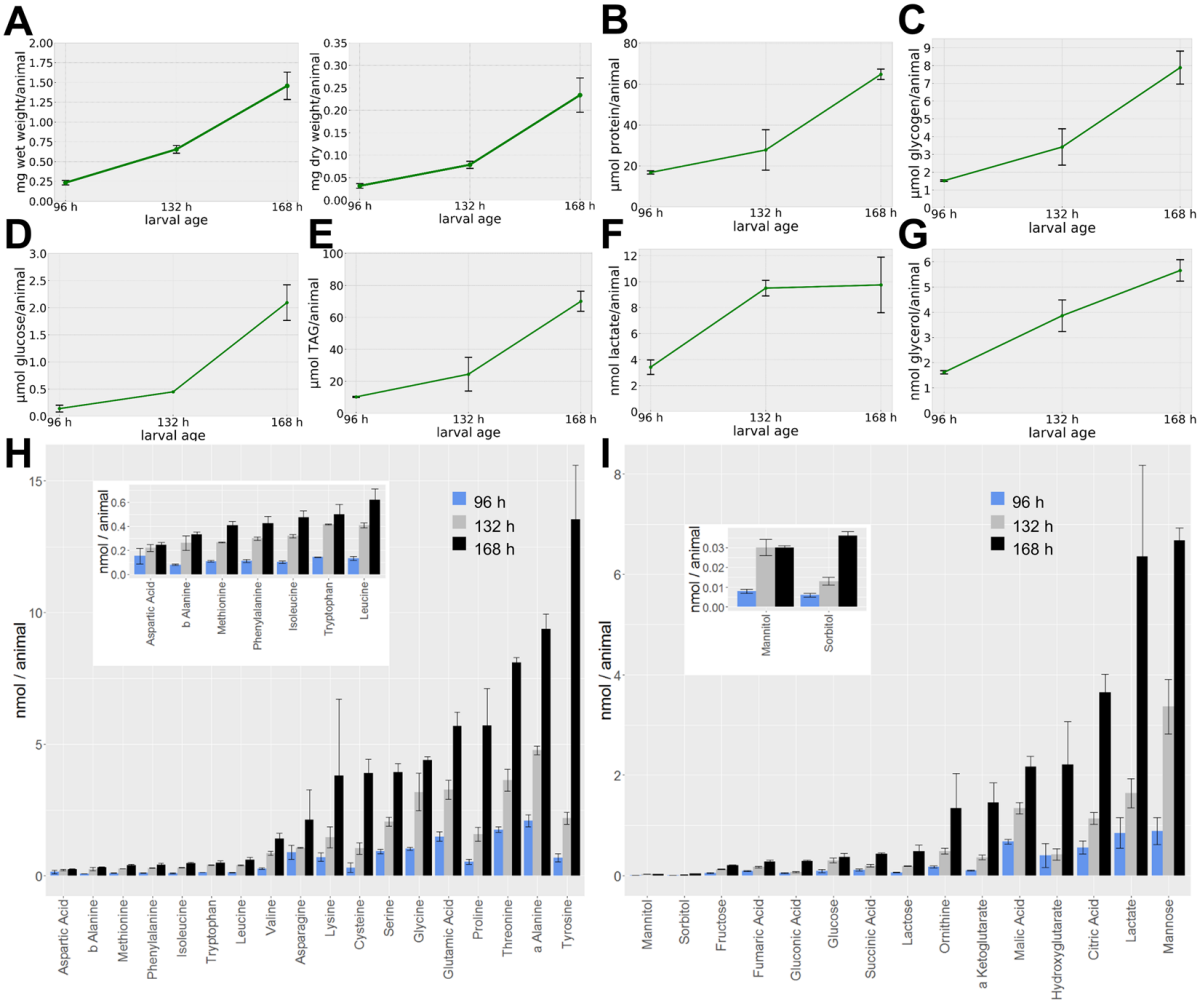
Growth and metabolic profiling of the larval developmental of *Drosophila melanogaster* 96, 132, and 168 hours after egg laying. (**A**) Wet weight (left plot) and dry weight (right plot) measurements of larvae at the indicated developmental time points. (**B**–**G**) Absolute quantification of protein, glycogen, glucose, triglyceride (TAG), lactate, and glycerol levels. The data represent mean values ± standard deviation normalized to the amount of animals per sample of at least triplicate measurements. (**H**,**I**) GC-MS metabolomics measurements of proteinogenic amino acids (**H**) and different metabolites (**I**) of *Drosophila* larval extracts from the indicated time points. Insets in (**H**,**I**) provide a zoom-in view on the low-abundant metabolite data. Metabolites were quantified using five point calibration curves (see methods, Fig. S3, and Table S2) and sorted for increasing abundance during the 168 hours time point.

**Figure 5:**
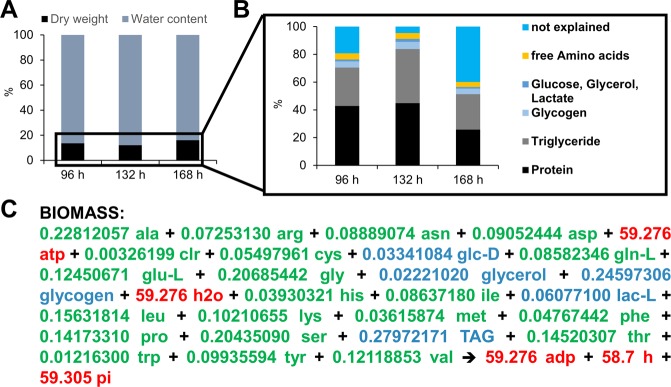
*Drosophila* body composition and biomass function. (**A**) Water content of *Drosophila* larvae raised on the HD during the three investigated time points. The black part of the stacked bar plots shows the dry mass of the larvae. (**B**) Our combined targeted and GC-MS metabolite measurements explain on average about 79% of the dry mass. Triglycerides and protein are the main contributors to dry mass. (**C**) *Drosophila melanogaster* biomass function based on our experimental data and literature. Green values indicate indices based on the GC-MS measurements and on the absolute biochemical metabolite quantifications, blue indices are based on the absolute biochemical metabolite quantifications. Red indices are based on information from the yeast whole-genome FBA model iMM904^32^.

Given our aim to investigate growth and resource allocation, we established the parameters of our model by incorporating experimental data. For this, we grew wildtype Oregon-R *Drosophila* animals on the HD. The complete larval development (until prepupae emerge) appears quasi linear and takes on the HD about 170 hours. In order to follow the development and metabolite profile over time, we collected larvae at three equally spaced time points during development (96, 132 and 168 hours after egg laying; AEL). To determine growth progression over time, we measured the wet and dry weight (Fig. 4A) as well as different size parameters (Fig. S2) of the larvae at the different time points. Larval weight increased almost linear over time (Fig. 4A). The water content was stable with values between 85 and 89% (Fig. 5A) and unaffected by alterations of the food composition (data not shown). For all time points, we performed absolute quantifications of free protein, glycogen, glucose, triacylglycerol (TAG), lactate, and glycerol (Fig. 4B–G) levels according to established protocols^31^ (see Table S2). Further, we quantified various metabolites of the central carbon metabolism as well as free amino acids by GC-MS metabolomics measurements and external standard curves (Figs 4H,I, S3 and Table S2). Most per animal normalized measurements increased over time, as expected (Fig. 4). Only lactate levels reached a plateau after 132 hours of development. All in all, our measurements explained on average 79% (for 96 h AEL: ~81%, for 132 h AEL: ~96% and for 168 h AEL: ~60%) of the total dry weight with proteins and TAGs being the major contributors (Fig. 5B). Of course, we can not rule out that the larval midgut contained metabolites from the HD, which we also included in our measurements. Yet, while none of our targeted metabolite measurements covered compounds present in the HD, those represented the most abundant constituents of the larval biomass. The metabolites quantified by the GC-MS metabolomics strategy – which covered also metabolites present in the HD as e.g. the singular amino acids – were only present in minute amounts. Thus, our measurements covered a large part of the body composition and should provide a sufficient approximation to model larval growth.

In order to perform growth rate simulations, we formulated a biomass function based on the previously reported yeast biomass function of the model iMM904^32^. Yet, from the original yeast biomass function, we only utilized the value for the growth associated maintenance (GAM) costs, as this measure is difficult if not impossible to obtain for a multicellular organism. The other coefficients of the biomass function are based on our own measurements (Fig. 5C and methods section). Next, we used the HD food ingredients as constraints for the model solution procedure. Although we already knew the exact composition of the food, we still needed to approximate the amount of food consumed by a single larva over time. Given that the measurement of the amount of internalized solid food is difficult, we decided to follow a theoretical approach (for details see methods section). For this purpose, we used data available for the average number of mouth hook movements (“bites”) per minute of larvae. The bites per minute only show low variability across different food compositions^33,34^, suggesting that this assumption is reasonable. Further, we approximated the volume of the mouth of the larva based on previous^35^ and own measurements (Fig. S2; approximated volume of the mouth = 0.011 mm^3^). Calculations based on both parameters resulted in an amount of 0.064 g/h food consumed per hour. We used this approximated food intake amount to calculate for each food ingredient the maximum amount consumed per hour. Of course, the calculated amount of consumed food is likely a prominent overestimation. For example, the larvae will most likely not fill their mouth completely with every bite. Further, not all food ingredients passage the gut barrier with 100% efficiency and at this point, we do not know the resorption rate for the different nutrients. Thus, we sought to identify a correction factor to limit the nutrient influxes to a reasonable level. For this purpose, we solved the FBA-model with a wide range of diminishing correction factors (Fig. S4) and compared the resulting calculated growth rates with our experimentally determined growth rate of approximately 8.8% dry weight increase per hour (see methods section). In this way, we identified a correction factor of the calculated food and thus nutritional uptake of about 12.2%. So far, little data concerning the food conversion and uptake rates of insects are available. Yet, Waldbauer *et al*. measured the efficiency of conversion of ingested food to body substance for various *Lepidoptera* species and found conversion rates ranging from 2 to 38%^36^. Our theory-derived value of 12.2% thus is in the range of the experimentally determined values of other insects.

### Model verification

**Figure 6:**
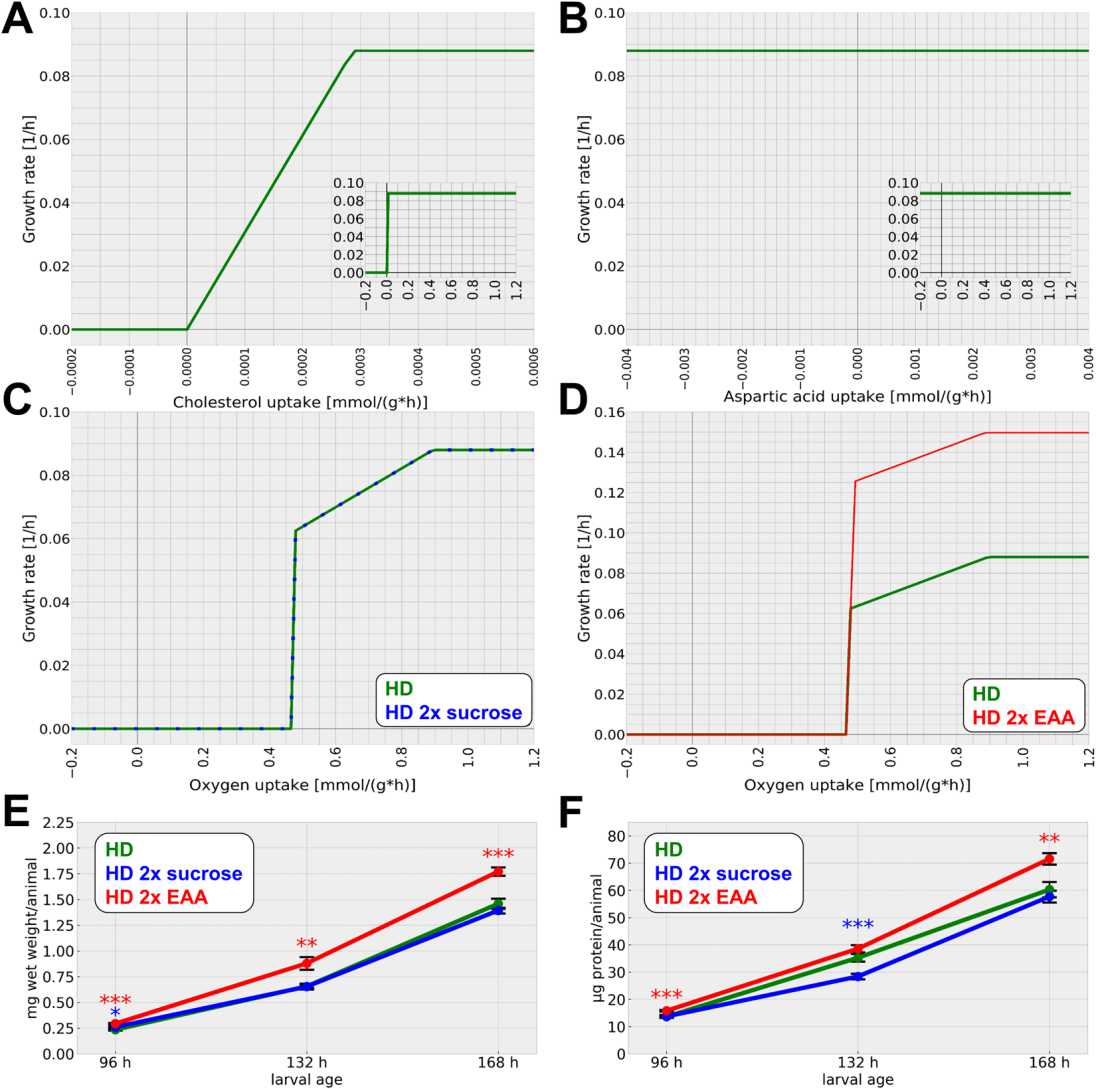
Model verification, predicted growth and comparison to real life. (**A**–**D**) Modeled growth rate in response to different input parameter variations. Negative uptake rates correspond to an excretion. (**A**) A certain level of cholesterol is needed for optimal growth while less result in a suboptimal growth phase. The zoom-in represents a larger cholesterol uptake flux range. (**B**) Levels of the non-essential aspartic amino acid do not affect biomass production. The zoom-in represents a larger aspartic acid uptake flux range. (**C**) Oxygen levels need to surpass a threshold to allow biomass production. In the following, the biomass production increases until it reaches a plateau. Increased sucrose levels (blue color) do not alter biomass production as compared to the standard HD (green color). (**D**) A doubling of the amount of essential amino acids (EAA) increases the biomass production (red color) as compared to the standard HD (green color). (**E,F**) Experimental testing of our model-based predictions. Animals were either reared on HD (green), HD containing the double amount of sucrose (blue) or the double amount of EAA (red). The wet weight (**E**) and protein (**F**) content of the larvae was measured 96, 132, and 168 hours after egg laying. While the altered sugar content did not affect the growth rate, the addition of more EAA resulted in a higher growth rate (**E**). The protein measurements show similar results (**F**). Measurements in (**E,F**) represent the mean values of three biologically independent experiments. Each experiment consisted of quadruplicate samples. Whiskers represent the standard error of the mean (SEM). Please note that the wet weight data for the HD is identical with the one shown in Fig. 4A. Statistical significance was tested by an unpaired two-sample T-Test for each time point. Significance levels are: *p < 0.05, **p < 0.01, ***p < 0.001.

The first step in our model verification procedure was to test whether it operates in a reasonable manner and whether it recapitulates known behaviors of the fly system, in contrast to e.g. the computer-generated model mentioned above. *Drosophila*, for example, is sterol auxotroph^27^. We built our model to recapitulate this behavior and indeed a steep increase in the growth rate for positive, non-zero sterol uptake rates is visible (Fig. 6A). In contrast, the amount of the non-essential amino acid aspartic acid had no effect on the growth rate, as expected (Fig. 6B).

As a next step, we performed more complex simulations. First, we investigated growth in response to varying oxygen levels. Here, a certain minimal oxygen influx was needed to support suboptimal growth before increasing oxygen levels resulted in a plateau of the growth rate (Fig. 6C). As a test for the predictive power of our system, we decided to test next whether we could predict the impact of diet alterations on the growth and metabolism. While a reduction of certain nutrients would have been possible, we decided to rather test for a possible limitation of certain nutrients given that the growth of the larvae was on the HD much slower as compared to a complex and rich diet. Based on the growth properties of the animals, the HD in fact is classified as a minimal medium, which was designed to mirror dietary restriction characteristics^26^. Thus, we increased either the amount of dietary sugar or essential amino acids that was possible to enter the model. A doubling of the sucrose input limit had no effect on the calculated growth rate (Fig. 6C). However, when we doubled the amount of essential amino acids (EAA) potentially entering the system, the predicted growth rate prominently increased (Fig. 6D). We subsequently tested our modeling predictions by performing corresponding experiments. Larvae reared on a HD containing the double amount of sucrose (“2x sucrose food”) indeed did not show an increased growth-associated weight gain as compared to larvae raised on the standard HD (Fig. 6E). Protein levels were even lower than the ones of the control animals at the middle time point (Fig. 6F). Doubling the amount of EAA (“2x EAA food”), however, indeed resulted in a higher growth rate (Fig. 6E) as well as higher amounts of protein (Fig. 6F). Thus, our modeling data are consistent with the experimental results, which suggests a high predictive power of the model.

### Modeling resource allocation

**Figure 7:**
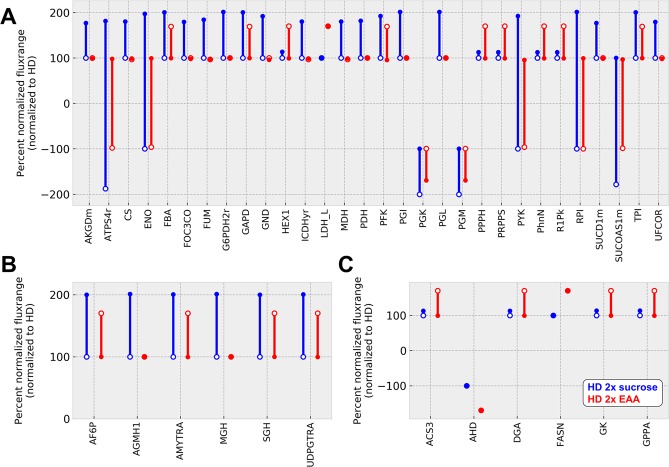
Modeling *Drosophila* larval resource allocation. The figure shows the results of the percent normalized flux variability analysis results for the simulations based on the HD with 2x sucrose (blue) and HD with 2x EAA (red), respectively. In brief, we percent normalized the values of the minimal (open circles) and maximal (filled circles) fluxes on the basis of the results for the standard HD. The sign of the flux percentage indicates the reaction direction, where a positive sign indicates the forward reaction, a negative sign indicates the backwards reaction and values spanning both signs represent reversible reactions. (**A**) Barplot of the central carbon metabolism flux change of the model solutions for the comparisons HD and HD with 2x sucrose (blue) or HD and HD with 2x EAA (red), respectively. (**B**) Flux change for the sucrose metabolism (description as in (**A**)). (**C**) Flux change for the lipid metabolism (description as in (**A**)). Each reaction description can be found in Table S1. Flux variability analysis results are given in Table S3 and the normalized flux data are presented in Table S4.

As a next step, we investigated whether the model could recapitulate resource allocation differences driven by the increase of sucrose or EAA in the HD. Therefore, we performed a flux variability analysis for our model with the given elevated maximum input limits (Table S3). Subsequently, we percent normalized the maximum and minimum flux values obtained for the 2x sucrose or 2x EAA food, respectively, based on the flux variability values of the standard HD (Table S4). Further, we split the metabolic reactions into functional groups and plotted selected reactions with altered fluxes (Fig. 7). On the simulated 2x sucrose diet, most reactions of the central carbon metabolism (Fig. 7A) showed a prominently increased maximum flux rate (e.g. FBA = fructose-bisphosphate aldolase, GAPD = glyceraldehye-3-P dehydrogenase, PFK = phosphofructokinase, PGI = glucose-6-P isomerase). On the simulated 2x EAA diet, however, most maximum flux rates did not change, and only the lower flux rate bounds were increased, thus resulting in a more narrow range of possible flux variations (Fig. 7A). For few reactions, the diet alterations resulted in opposite flux changes (HEX1 = hexokinase 1, LDH_L = l-lactate dehydrogenase, PPPH = diphosphate phosphohydrolase, PRPPS = ribose-phosphate diphosphokinase, PhnN = ribose 1,5-bisphosphate phosphokinase, R1Pk = Ribose 1-phosphokinase). The diphosphate phosphohydrolase (PPPH) flux showed a largely increased minimal flux on the simulated 2x EAA diet (Fig. 7A). Diphosphate phosphohydrolase activity takes place very early in the lipid degradation, as it acts as the force to activate fatty acids for the beta-oxidation^37^. The higher minimal flux following the elevated EAA input, suggested an enhanced rate of lipid activation, which potentially fuels the elevated growth. Further, the increased flux of the lactate dehydrogenase suggested increased lactate levels of animals reared on the diet with 2x EAA.

As expected, fluxes of reactions involved in sucrose metabolism (AF6P = ATP:D-fructose 6-phosphotransferase, AGMH1 = 1,4-alpha-D-Glucan maltohydrolase AMYTRA = 1,4-alpha-D-Glucan:1,4-alpha-D-glucan 6-alpha-D-(1,4-alpha-D-glucano)-transferase, MGH = maltose glucohydrolase SGH = sucrose glucohydrolase, UDPGTRA = UDP-glucose:glycogen 4-alpha-D-glucosyltransferase) increased prominently if the sucrose input was increased (Fig. 7B). The simulation of elevated EAA levels resulted in increased minimal fluxes of enzymes involved in lipid metabolism (ACS3 = acyl coenzyme A synthetase, DGA = 1,2-diacylglycerol acyltransferase, GK = glycerol kinase, GPPA = alpha-glycerophosphate acyltransferase), as well as situations where both the lower and upper flux limits were elevated (FASN = fatty-acid synthase), which suggest elevated lipid storage levels as a result (Fig. 7C).

**Figure 8:**
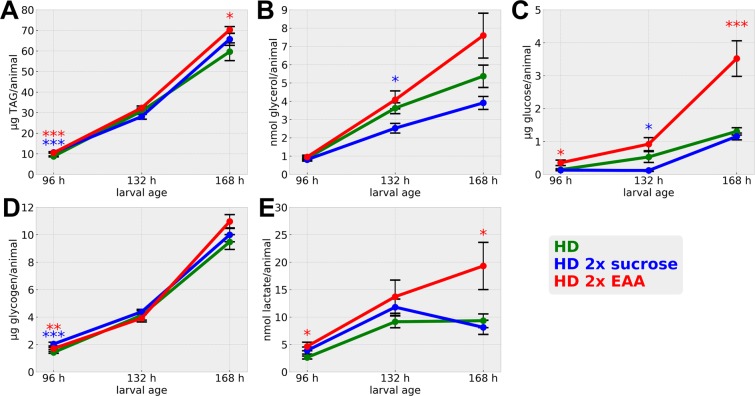
Metabolic profiling of the larval developmental of *Drosophila melanogaster* 96, 132, and 168 hours after egg laying. (**A**–**E**) Absolute quantification of triglyceride (TAG), glycerol, glucose, glycogen, and lactate levels. Measurements in (**A**–**E**) represent the mean values of three biologically independent experiments. Each experiment consisted of quadruplicate samples. Whiskers represent the standard error of the mean (SEM). Statistical significance was tested by an unpaired two-sample T-Test for each time point. Significance levels are: *p < 0.05, **p < 0.01, ***p < 0.001.

When we performed corresponding metabolite measurements with animals raised under the different growth conditions, TAG levels indeed showed a modest, but significant, increase following growth on the 2x EAA diet (Fig. 8A). Free glycerol levels showed a larger amount of variation (Fig. 8B). Yet, the trends clearly differed in response to the varying diet compositions. Increased dietary sugar levels resulted in lower levels of free glycerol, whereas increased amounts of EAA resulted in an on average increased free glycerol levels indicative of elevated lipolytic and/or lipogenic activity. Intuitively, we expected an altered sugar content of the animals raised on the 2x sucrose diet. Yet, we did not detect any prominent differences (Fig. 8C), which seems to be in line with the modeling results, which indicate a larger flux of the glycolysis reactions with a simultaneous activation of the complete TCA cycle or the reactions involved in oxidative phosphorylation (Fig. 7A). This increase of flux values suggest that the metabolism of *Drosophila* activates a metabolic program for an overflow metabolism perhaps associated with a larger burning or excretion of sugars. The 2x EAA diet, however, resulted in a significantly higher glucose content of the animals (Fig. 8C), which appears to be in line as the higher flux rate of diphosphate phosphohydrolase or fatty-acid synthase suggest a higher beta-oxidation besides an increased lipid storage predicted by the model. Glycogen levels were mostly unaffected by the altered nutritional conditions (Fig. 8D). Lactate showed under basal HD growth conditions a plateau 132 hours after egg laying (Figs 4F, 8E). If additional sucrose was present, lactate levels even decreased 132 hours after egg laying (Fig. 8E). The 2x EAA diet yet resulted in statistically increased lactate levels (Fig. 8E), which again is in line with our modeling results, which suggested an increased lactate dehydrogenase flux (Fig. 7A). Thus, we also could largely align our modeled flux values with the corresponding experimental data.

## Discussion

Flux balance analyses (FBA) with metabolic networks of heterotrophic multicellular organisms usually appears complicated due to the difficulty of identifying a clear-cut objective function. Further, the various cell types of complex organisms, with their signature gene expression profiles and distinct metabolic and functional tasks, complicate modeling approaches even more. To tackle these difficulties, methods and strategies were designed^38^, as for example, to model the metabolic flow in the context of gene regulation or other advanced constraints such as dynamic changes. Here, we decided to use a top-down modeling approach focusing on a generalized and averaged simple model of the growing larva, which we identified as a suitable system for FBA given its clear-cut objective function. We used this simplified model given that many details of the overall organismic physiology are still unclear. Therefore, we rationalized that adding uncertain information might rather act detrimental as compared to a simplified model, which could better catch the more general schemes. A benefit from starting our model mostly from scratch was that we could pay special attention on avoiding dead-end and blocked-reactions (Fig. 3), as well as to assure the biological feasibility of the subsequent FBA modeling results. Our resulting model is to the best of our knowledge the currently most advanced metabolic network of *Drosophila* metabolism and we provide it together with the code to perform FBA analyses in different ready-to-use formats to the community (see methods section). Our initial rationale that a simple model might be already useful appears valid, given that it is already able to recapitulate basic biology (Fig. 6) and successfully predicted the impact of dietary alterations on the larval growth and metabolism (Figs 6–8). In the future, the refinement of the model by e.g. incorporating gene regulation or a dynamic modeling^38^, potentially further enhances the predictive power of the model. While these enhancements will be likely less important for the modeling of a general behavior of the system – and thus general resource allocation questions as there are still many uncertainties resulting in a high number of degrees of freedom –, they will facilitate the studying of new questions. For example, it will be interesting to investigate the interplay between different organs, as well as to study inter-organismal metabolic connections such as between the host and its gut microbiome constituents or host-symbiont/host-parasite mutualism. *Drosophila* is an exquisite model for such kind of studies given its well characterized and relatively simple gut microbiome^39^. Further, the mutualism between insects and the endosymbiont *Wolbachia*, which can also act as pathogen, is well described^40,41^. In *Aedes aegypti*, an impact of an infection with the pathogenic *Wolbachia* strain *wMelPop*, for example, was recently shown to affect the TAG and cholesterol metabolism of the host^42^. Further, the time-resolved analysis of metabolite amounts as well as the consequences of gene dosage and protein activity alterations, will be interesting avenues to follow.

Our lack of knowledge concerning the energy costs associated with growth and non-growth associated processes (often referred to as GAM – growth associated maintenance, and NGAM – non-growth associated maintenance costs) might appear as a weak spot of our approach. So far, we determined the NGAM values in an iterative process based on the oxygen consumption rate of *Drosophila* S2R+ cells^43^ (methods section and Fig. S5) and used the GAM values from a yeast model^32^, as an experimental estimation of the values for *Drosophila* is difficult. Our approach appears legitimate, at least at the current point of time, as simulations testing a wide array of different GAM and NGAM values with our model demonstrated only a limited impact on the biomass production (Fig. S6 and Interactive Supplementary Fig. 2). The impact of GAM and NGAM uncertainties on the resource allocation problem might be bigger. The size of the linear programming solution space decreases with increasing GAM and/or NGAM values, as fluxes have to compensate for the increased energy requirements as for example the rate of oxygen consumption. Thus, the future estimation of the real GAM and NGAM values for larvae under different physiological conditions is a challenging, yet valuable goal.

We parameterized our model using absolute enzyme-based biochemical quantifications as well as via GC-MS based metabolomics measurements (Figs 4–6 and Table S2). Overall, our measurements explained a large portion of the dry weight of the animals (Fig. 5B). The prominent drop of explained dry weight at 168 hours after egg laying (60% explained dry mass versus 81 or 95% (96 or 132 hours after egg laying, respectively)) is intriguing. Given that despite of lactate all metabolites measured by us increased over time (Figs 4 and 8), this result suggests that the production of another metabolite not measured by us is increasing dramatically during this growth phase. Candidates are, for example, nucleic acids or components of the cuticle. In support of this notion, we noted an elevated slope of the larval weight gain from the second to the last time point (Fig. 4A), whereas size increase on the HD rather stalled during this phase (Fig. S2B–D).

The use of the chemically defined HD significantly facilitated the connection of the biological data to the modeling both under basal (Figs 4–8) as well as dietary altered (Figs 4, 6 and 8) conditions. The presence of the chemically defined food is a big advantage as compared to complex and ill-defined food compositions, as uncertainties in terms of the diet obfuscates defining the real inputs entering the system in the experiment as well as the model. An obvious point for optimization is our lack of knowledge concerning the exact amounts of food consumed and metabolite resorption rates. The mouth hook contraction values used by us, for example, could in theory vary in response to different diets. Several reports, however, suggested only a limited impact of the diet on the mouth hook contraction frequencies^33,34^. Nevertheless, in the future, methods that are more sophisticated should be used to eliminate this shortcoming using e.g. radiolabeled tracer experiments, which will also allow the direct estimation of metabolite flux rates. These in turn will allow a much better comparison to the predicted flux rates as our endpoint measurements. Another point to consider is that the HD represents a minimal medium, which results in a slowed down growth. Here, we started to investigate which nutrients might act as growth limiting factors and our strategy appears to be successful in predicting nutrients allowing a faster growth. For the investigation of resource allocation and optimality principles, the slowed-down growth appears less problematic, given that optimality of resource allocation is situation dependent, and thus an inadequate diet can also be utilized in an optimal manner.

Investigations concerning the minimal nutritional requirements of an organism, as well as concerning the impact of nutritional alterations on the physiology of an organism, is an active field of research^44–46^. Our early results with the predictions of the impact of altering sucrose or EAA levels in the food are particularly promising. They suggest that simulations with FlySilico should facilitate the identification of suitable parameter ranges for experiments targeting e.g. the nutritional requirements of larvae and flies or the impact of diet alterations on the metabolic and/or growth phenotype. The future FlySilico-based investigations concerning the impact of varying amounts of essential and branched chain amino acids on growth processes, life history traits such as fecundity, ageing related diseases and cancer will be exciting as these aspects gained a lot of attention recently^47–50^.

*Drosophila* larval growth is marked by an impressive increase in size and mass. This expansion is necessary for a successful completion of metamorphosis, which involves a drastic remodeling of body structures and a food intake cessation. Therefore, the larval development is subject to hard biological constraints. Still, the organism can react to fluctuations in the quality or quantity of food by adjusting the rate of development and resource allocation, e.g. by channeling less nutrients into storage forms^15^. Our experiments, where we raised animals on either standard HD or HD supplemented with additional sucrose or EAA (Figs 4, 6 and 8), demonstrate this plasticity. Further, our model predictions correctly identified the growth limiting nutritional parameters and revealed flux differences, which relate to the observed metabolic changes based on the diet alterations. The precision of the model predictions will increase with further improvements as outlined above. Intriguingly, the metabolic adaptation to altered nutritional conditions are not limited to insects, but have also been described e.g. for mammals^20^. Thus, future investigations targeting other species are possible to test for a possible generalization of our findings.

An important aspect is that solving the flux balance model is by definition following optimality principles. Given that our relatively simple model already was able to result in correct predictions suggests that *Drosophila* resource allocation is operating in a quasi-optimal state. Future studies with additional parameter variations (e.g. cost functions for protein and DNA synthesis and distinguishing different type of organs or tissues^38^) and incorporation of additional fly genotypes and perhaps single gene mutations will help to further elucidate this intriguing possibility.

## Materials and Methods

### Drosophila fly stocks and rearing

For all our experiments, we used the wild type Oregon-R fly strain reared under standard culture conditions (25 °C, 12 h light-dark rhythm and 60–70% humidity).

### Chemically defined fly medium

The chemically defined (holidic) medium (*Holidic diet*; HD) was introduced in^26^. Animals developing on HD are viable, fertile, and have no aberrant phenotypes, although the development is slowed-down and the HD is thus classified a minimal medium. Food was prepared according to the instructions of the Piper *et al*. publication with use of the Yeast-like amino acid composition (“Yaa”). For our perturbation experiments we either added the double amount of sucrose (2x sucrose) or the double amount of essential amino acids (2x EAA).

### Larvae collection procedure

In order to minimize a possible impact of environmental effects, we kept the parental density constant with 15 male flies and 30 female flies per vial. These adult flies were kept on a standard complex cornmeal diet (per 100 mL: 0.5 g agar (Becton Dickinson, 214010), 7.1 g polenta (Verival, Pronurel Bio, 265250), 0.95 g soy flour (Bauck Hof, Amazon.de, B004RG3C0I), 1.68 g yeast (Bruggeman, lieferello.de, 14874413), 4 g treacle (Original Grafschafer Goldsaf, lieferello.de, 10231869), 4.5 g malt extract (Demeter, Amazon.de, B00GU029LW), 0.45 mL propionic acid (Acros Organics, 220130010, CAS 79094) and 1.5 mL nipagin (Sigma-Aldrich, H3647-100G) (1:10 stock solution in 70% Ethanol; Riedel-de Haën, 16202S-1L, CAS 64-17-5) before we transferred them to the chemically defined medium to allow oviposition. After six hours we discarded the parental flies to have a defined time period for the egg-laying. As the maximum time point for collecting larvae we used 168 h after egg laying as afterwards the larvae start to pupariate on the HD. Based on this terminal point, we added two equally spaced time points earlier in development (132 h and 96 h after egg laying, respectively) as growth is quasi linear.

The possibility that larval growth and metabolism is showing a sexual dimorphism appeared intriguing. Pilot experiments using animals reared on the holidic or a standard diet, however, showed that at the latest timepoint used by us female and male larvae do not significantly differ in terms of the size, the triglyceride, glucose or glycogen levels (data not shown). Thus, we did not consider the sex of the animals during our experimental timeframe a prominent factor and therefore collected unsexed larvae 96 h, 132 h and 168 h after egg laying and washed them in PBS with 0.1% Tween-20 (PBT) for the weight measurements and metabolic assays or in HPLC-graded water for the GC-MS analytics. We used quadruplicates for every condition and collected 25 larvae (96 h) or eight larvae (132 h, 168 h) for the GC-MS and the metabolic assays. For the dry and wet weight measurements, we collected 100 animals from the 96 h time point and 40 animals from the 132 h and 168 h time points, respectively.

### Wet and dry weight measurements

For the determination of the wet weight, we transferred the washed larvae into pre-weighed 1.5 mL tubes and weighed them on an analytical scale. The animals were then snap-frozen in liquid nitrogen and dried in an oven at 60 °C with the tube lids open. After 24 h we measured again the weight ( = dry weight) and calculated the water content by subtraction of the dry weight from the wet weight.

### Larval size measurements

For the larval size measurements, we collected at the indicated time points the animals in ice cold PBS to minimize their movements and to ensure their elongation. Subsequently, we recorded images with a Zeiss SteREO Discovery.V8 dissection microscope, which were analyzed with the Zeiss Zen Software (Zen 2.3 lite – blue edition). For each larva, we measured the area, the length and its width. In total, we performed three biologically independent experiments and measured 20 to 30 animals per repetition.

### Biochemical measurements

All targeted biochemical measurements were essentially carried out as described in^31^. We collected the larvae from three different time points and snap-froze the animals in liquid nitrogen before storage at −80 °C. For the homogenization, we used 1 mL 0.05% Tween 20 in water in 2 mL screw-cap tubes and a Fast Prep FP120 machine (Bio101 Savant). After homogenization and heat-inactivation for 5 minutes at 70 °C, the supernatant was transferred to 1.5 mL tubes as a reservoir for the metabolic assays, which were performed in 96-well plates. We normalized each measurement to the amount of animals per sample.

#### Protein

The free protein content was measured using the Pierce BCA assay kit (Life Technologies) according to the manufacturer’s instructions. We used bovine serum albumin (BSA) as a standard to determine the protein content of the samples. The 0.05% Tween-20 used by us in the homogenization procedure most likely was not sufficient to solubilize also integral membrane proteins. Thus, the protein amounts per animal might represent an underestimation of the real total protein content.

#### Triglycerides (TAG)

For the determination of the triglyceride levels in the samples, we used the Triglycerides Reagent (Thermo Scientific). We transferred 50 µL of the samples and a serial dilution (1:2 in 0.05% Tween 20 in water) of the glycerol standard (Sigma Aldrich) to a 96-well plate and added 200 µL of the Triglycerides reagent. The samples and the standard were incubated 45 minutes at 37 °C and the absorbance was read at 510 nm.

#### Glycerol

The glycerol content of the samples was determined using the Glycerol Assay Kit (Sigma-Aldrich). We followed the manufacturer’s instruction for fluorometric measurements.

#### Glucose and Glycogen

For the determination of glucose and glycogen, we used the GO Assay Reagent (Sigma-Aldrich) and a modified form of a protocol described in^51^. For both measurements, we transferred 30 µL of the undiluted samples and the standards to a 96-well plate. We added 100 µL GO reagent to measure free glucose and 100 µL GO reagent with amyloglucosidase (1 µL per 1 mL GO reagent) to measure the total glucose content (free glucose plus glucose liberated from the glycogen). After 60 minutes incubation by 37 °C we stopped the reactions by adding 100 µL 12 N H_2_SO_4_ and measured the absorbance at 540 nm. We calculated the glycogen content by subtraction of the free glucose from the total glucose content.

#### Lactate

For the quantification of lactate, we used the Lactate Assay Kit (Biovision). We transferred 50 µL of the pre-diluted samples (1:50) to a 96 well plate and followed then the manufacturer’s instruction for fluorometric measurements.

#### Cholesterol

Measurements were performed as described in^52^.

### GC-MS measurements

Metabolites were extracted using 105 µL chloroform and 245 µL methanol. After incubation for 1 h at −20 °C we added 560 µL HPLC grade water twice. The samples were centrifuged for two minutes at high speed in a table top centrifuge at 4 °C and the aqueous phases were collected for the GC-MS measurements (in total about 1.3 mL).

For the metabolite analysis a gas chromatography – mass spectrometry (GC-MS) system (7200 GC-QTOF from Agilent) was used as described in^53^. The data were analyzed with the Mass Hunter Software (Agilent). For absolute quantifications, we used five different dilutions of the standard mix (resulting in effective metabolite concentrations: 1 µM, 5 µM, 10 µM, 15 µM and 20 µM; Fig. S3) and calculated for each metabolite a standard curve which we used to determine the amount of the respective metabolite in our samples.

### Network reconstruction

For the *in silico* reconstruction of *Drosophila melanogaster* growth and metabolism we focused on core metabolic pathways required to metabolize the HD ingredients, and used the cameo package for the Python programming language^54^. The Yeast iMM904 model from the BiGG data base^32^ and a previously published *Drosophila* model for hypoxia investigations^30^ served as starting points for our network reconstruction. First, we incorporated major metabolic pathways of the carbohydrate metabolism (including glycolysis, gluconeogenesis, tricarboxylic acid (TCA) cycle and pyruvate metabolism), the lipid metabolism (with a focus on the glycerolipid metabolism), the energy liberating metabolic reactions (e.g. oxidative phosphorylation) and anaplerotic reactions. Subsequently, we successively integrated metabolic reactions necessary to metabolize the HD ingredients, such as amino acid metabolic pathways (including e.g. glycine, threonine, cysteine, and phenylalanine metabolism), pathways of vitamin synthesis (e.g. folate and riboflavin) and various pathways needed for the transport and synthesis of cofactors. For the initial version of FlySilico we only focused on three different compartments (extracellular, cellular and mitochondrial) and adapted the transport reactions accordingly. We manually curated all reactions by cross-validation with multiple resources (BioCyc - https://biocyc.org/ ^55^, BRENDA - https://www.brenda-enzymes.org/ ^56^, ChEBI - http://www.ebi.ac.uk/chebi/init.do ^57^, KEGG - http://www.genome.jp/kegg/ ^58^, PubChem - https://pubchem.ncbi.nlm.nih.gov/ ^59^, BiGG - http://bigg.ucsd.edu/ ^60^, and FlyBase - http://flybase.org/ ^61^) and paid special attention on the biochemical pathways and the genetics of *Drosophila melanogaster*. We attached to each reaction a confidence score based on evidence of sequence, physiological, genetic or biochemical data (as previously suggested by e.g.^62^). Reactions required for the modeling, but without any evidence of correctness, received the lowest confidence scores. FlySilico version 1.0 covers 293 metabolites and 363 reactions. Supplemental Table S1 summarizes all pathways, reactions and metabolites present in the model.

### Constraint-based modeling

After the reconstruction process, the coefficients of the mass-balanced reactions form a mathematical representation as stoichiometric matrix S. The constraint-based modeling approach follows equation:

1 $$\frac{{\rm{dx}}}{{\rm{dt}}}={\bf{S}}\cdot {\boldsymbol{v}}=0$$

with

2 $${{\boldsymbol{\alpha }}}_{{\boldsymbol{i}}}\le 0\le {{\boldsymbol{\beta }}}_{{\boldsymbol{i}}}$$

where x is a vector with all metabolites, S is the stoichiometric matrix and v is a vector with all fluxes under steady state conditions. The lower (α_i_) and upper (β_i_) bounds to each flux v_i_ impose additional constraints to the system. The null space of S includes any v that satisfies the solution under this steady state assumption with the given constraints. The model is solved by optimizing the system for a given objective function, i.e. the primary goal of an organism (such as biomass production for fast growing unicellular organisms as for example *E*. *coli*), using linear programming. A detailed explanation of constraint-based modeling, flux balance analysis and linear programming is provided e.g. in:^63^ or^64^. For our model solutions we compared solutions allowing loops as well as loopless^65,66^ variants (Fig. S7). The loopless solution reflects the biology better, and thus we used this method throughout the study unless otherwise noted.

### Flux variability analysis

Flux variability analysis (FVA^67^) is a computational method to identify the maximum and minimum fluxes of reactions from a given network while it preserves a certain network state (e.g. maximum biomass production rate). FVA solves two optimization problems for each reaction *v*_*i*_ after solving a given objective function.

3 $$\begin{array}{c}ma{x}_{v}/mi{n}_{v}\,{v}_{i}\\ {\rm{subject}}\,{\rm{to}}\,{\bf{S}}\cdot {\boldsymbol{v}}=0\\ \,\,\,\,\,{{\boldsymbol{c}}}^{T}\,\ast \,{\boldsymbol{v}}\ge \gamma \,\ast \,{Z}_{0}\\ \,\,\,\,\,{{\boldsymbol{\alpha }}}_{{\boldsymbol{i}}}\le 0\le {{\boldsymbol{\beta }}}_{{\boldsymbol{i}}}\end{array}$$

where ${Z}_{0}=\,{{\boldsymbol{c}}}^{T}{{\boldsymbol{v}}}_{{\boldsymbol{o}}}$ is an optimal solution for the objective function, *γ* is a parameter which controls the optimality of the solution (suboptimal: $0\,\le \,\gamma \, < 1$; optimal: *γ* = 1), **c** is the vector which represents the linear objective function.

### Calculation of biomass and uptake rates

In order to identify an appropriate biomass function, we estimated the body composition of differently aged larvae by targeted absolute biochemical quantifications as well as GC-MS based metabolomics measurements. We reasoned that the main larval constituents are water, proteins, carbohydrates and storage lipids given that the latter two are the main storage forms fueling metamorphosis and that the larvae are filled up with the adipose-tissue like storage organ – called the fat body – which is the main storage site for storage lipids and glycogen. The water content could be easily measured by gravimetrics (see above) and on top of free protein, glucose, triglyceride and glycogen amounts, we also determined the levels of lactate and free glycerol by targeted biochemical assays (see above). Our GC-MS measurements further covered metabolites from the central carbon metabolism as well as almost all free amino acids.

Biomass functions usually cover each amino acid separately. Yet, our free protein measurements did not provide such fine-grained information. The GC-MS based metabolomics measurements resulted in the identification of free amino acid amounts; yet three amino acids (arginine, glutamine and histidine) were missing in our measurements. In order to approximate the levels of the different amino acids, we followed a bioinformatics strategy with the reasoning that the amino acid fractions would relate to the respective amino acid frequency across the *Drosophila* proteome. Thus, we first calculated the frequency of each of the twenty classical amino acids in the complete proteome of *Drosophila melanogaster* (http://www.uniprot.org/uniprot/?query=proteome:UP000000803). Figure S8 provides the calculated amino acid frequencies. We based the coefficients on the differences between the first and last time points investigated. Thus, for each measurement we calculated:

4 $${m}_{168h,Metabolite}-{m}_{96h,Metabolite}={m}_{\Delta ,Metabolite}$$

where ${m}_{\Delta ,Metabolit{e}_{i}}$ represents the weight difference of a metabolite *i* between 168 h and 96 h in $gram$, ${m}_{168h,Metabolit{e}_{i}}$ is the weight of the metabolite *i* at 168 h in $gram$, and ${m}_{96h,Metabolit{e}_{i}}$ is the weight of the metabolite *i* at 96 h in $gram$. For the amino acids, we could now calculate the individual amino acid weights with the help of the calculated amino acid frequencies:

5 $${m}_{\Delta ,Metabolit{e}_{i}}\hat{=}{m}_{A{A}_{i}}={m}_{\Delta ,Protein}\,\ast \,\,{f}_{A{A}_{i}}$$

where ${m}_{A{A}_{i}}$ shall be equivalent to ${m}_{\Delta ,Metabolit{e}_{i}}$ and represents the weight of amino acid *i* in $gram$, ${m}_{\Delta ,Protein}$ is the difference of weight of the protein in $gram$ which was calculated based on equation Eq. 4, and *f*_*AAi*_ which represents the frequency of amino acid *i* from Fig. S8. Metabolite weights from equations Eqs 4 and 5 enable the calculation of an assay-based coefficient for each metabolite for the biomass objective function:

6 $${x}_{Assay,Metabolit{e}_{i}}=\frac{\frac{{m}_{\Delta ,Metabolit{e}_{i}}}{{M}_{Metabolit{e}_{i}}}}{{m}_{\Delta ,dry}}=\,\frac{{n}_{Metabolit{e}_{i}}}{{m}_{\Delta ,dry}}$$

where ${x}_{Assay,Metabolit{e}_{i}}$ represents the coefficient of the metabolite based on assay data in $\frac{mmol}{g}$, ${M}_{Metabolit{e}_{i}}$ is the molar mass of metabolite *i* in $\frac{g}{mmol}$, ${m}_{\Delta ,dry}$ is the larval dry weight difference from 96 h till 168 h in *gram*, and ${n}_{Metabolit{e}_{i}}$ is the amount of metabolite $i$ in $mmol$.

The GC-MS analysis quantified free metabolite amounts. In order to calculate a GC-MS coefficient, we calculated the difference of metabolite amounts between 168 h and 96 h by the following equation:

7 $${n}_{\Delta ,Metabolit{e}_{i}}={n}_{168h,Metabolit{e}_{i}}-{n}_{96h,Metabolit{e}_{i}}$$

where ${n}_{\varDelta ,Metabolit{e}_{i}}$ represents the difference of amount between 168 h and 96 h from metabolite *i* in $mmol$, ${n}_{168h,Metabolit{e}_{i}}$ is the amount of the metabolite *i* at 168 h in $\,mmol$, and ${n}_{96h,Metabolit{e}_{i}}$ is the amount of the metabolite *i* at 96 h in $mmol$. We used the resulting metabolite amounts from equation Eq. 7 to calculate a GC-MS coefficient with the equation:

8 $${x}_{GCMS,Metabolit{e}_{i}}=\,\frac{{n}_{Metabolit{e}_{i}}}{{m}_{\Delta ,dry}}$$

where ${x}_{GCMS,Metabolit{e}_{i}}$ as the GC-MS coefficient of the metabolite *i* in $\frac{mmol}{g}$, ${m}_{\Delta ,dry}$ is the difference of dry weight between 168 h and 96 h in $gram$, and ${n}_{Metabolit{e}_{i}}$ is the amount of metabolite *i* in $mmol$.

Through equation Eqs 6 and 8 the biomass function coefficient can be calculated for all metabolites with the following equation:

9 $${x}_{Metabolit{e}_{i}}={x}_{Assay,Metabolit{e}_{i}}+{x}_{GCMS,Metabolit{e}_{i}}$$

where ${x}_{Metabolit{e}_{i}}$ is the biomass function coefficient of metabolite *i*, ${x}_{Assay,Metabolit{e}_{i}}$ as the assay-based coefficient of metabolite *i*, and ${x}_{GCMS,Metabolit{e}_{i}}$ as the GC-MS-based coefficient of metabolite *i*. All coefficients of Eq. 9 are in $\frac{mmol}{g}$. *Drosophila melanogaster* is cholesterol auxotroph^27,68^. To simulate the cholesterol auxotrophy of *Drosophila*, we included cholesterol in the biomass function based on measurements of cholesterol levels from larvae reared on HD (Fig. S9). The biomass coefficient of cholesterol was calculated according to the equation:

10 $${x}_{Cholesterol}=\,\frac{\frac{{c}_{Cholesterol,Protein}\,\ast \,{m}_{\Delta ,Protein}}{{M}_{Cholesterol}}}{{m}_{\Delta ,dry}}$$

where ${x}_{Cholesterol}$ is the biomass coefficient for cholesterol in $\frac{mmol}{g}$, ${c}_{Cholesterol,Protein}$ is the cholesterol mass per protein mass in $\frac{g}{g\,Protein}$, $\,{m}_{\Delta ,Protein}$ is the difference of weight of protein between 168 h and 96 h in $gram$, ${M}_{Cholesterol}$ is the molar mass of cholesterol in $\frac{g}{mmol}$, ${m}_{\Delta ,dry}$ is the difference of dry weight between 168 h and 96 h in $gram$. The mean value of the cholesterol mass per protein mass of all 3 time points (96 h, 132 h and 168 h) is ${c}_{Cholesterol,Protein}\approx 5.46\,\frac{ng}{\mu g\,Protein}$.

### Approximation of food intake

Given that the absolute quantification of the uptake of solid food by larvae is difficult, and that the measurement of the absorption rate and organismic distribution for each nutrient is close to impossible, we used a theoretical approximation of the food intake as a starting point for our modeling experiments. First, we calculated the maximum volume of the mouth cavity by approximating a cylindrical shape and taking length and diameter measurements of the differently aged larvae into account. We calculated the oral cavity volume according to equation:

11 $${V}_{mouth}=\pi \,\ast \,{r}^{2}\,\ast \,h$$

where *V*_*mouth*_ is the volume of the oral cavity in *mm*^3^, *π* is the mathematical constant, *r* is the radius of the oral cavity in *mm*, and *h* is the height of the oral cavity in *mm*.

Because the larva grows over time, we estimated the diameter of the larva as the mean of the width of time points 96 h and 168 h and assumed that the diameter of the oral cavity is about half of the larva diameter (the radius of the oral cavity thus is $r=0.118\,mm$). We took the height $h$ of the oral cavity from a publication from Alpatov^35^. Here, the height of the oral cavity is the average of the mean length from larval stages II and III with a value of $h=0.25\,mm$. Thus, the resulting oral cavity volume according to equation Eq. 11 is *v*_*Mouth*_ = 0.011 *mm*^2^.

We approximated the feeding rate using measures of the sclerite reactions per minute from^69^ $({f}_{Feed}=\frac{110}{min})$, with the assumptions that each sclerite reaction completely fills the oral cavity and that all food ingredients are homogeneously distributed. The dietary intake can thus be calculated using the following equation:

12 $${m}_{Dietary}={v}_{Mouth}\,\ast \,{f}_{Feed}\,\ast \,{\rho }_{holidic}$$

where *m*_*Dietary*_ is the dietary intake in $\frac{g}{h}$, *v*_*Mouth*_ is the oral cavity volume in *mm*^3^, *f*_*Feed*_ is the sclerite reactions per *h*, and ${\rho }_{holidic}$is the sum of the mass concentrations of the holdic diet ingredients in $\frac{g}{\mu L}$. Our calculated dietary intake is ${m}_{Dietary}=0.064\frac{g}{h}$.

Our calculation is of course an overestimation given that each sclerite reaction most likely does not fill the mouth volume completely and that the uptake from the gut is not 100% efficient. To account for this limitation, we introduced a correction factor *χ* based on our experimental data and simulations by an iterative process. In brief, we calculated all uptake rates with increasing values for the correction factor *χ* (from 0 to 0.20 in 0.001 steps) and used the different uptake rates to calculate the corresponding growth rate. We selected for *χ* the value where the calculated growth rate fitted the experimentally determined growth rate best (Fig. S4). We determined the experimental growth rate based on the dry weight measurements during the three time points. We calculated the growth rate between the first and last time point as:

13 $${\mu }_{BIOMASS,Exp}=\frac{\frac{{\bar{m}}_{t2}-{\bar{m}}_{t1}}{\Delta t}}{{\bar{m}}_{t1}}$$

As a result we obtained the experimental growth rate ${\mu }_{BIOMASS,Exp}=\frac{0.0882}{h}$ and for $\chi =0.122$ the predicted growth rate ${\mu }_{BIOMASS,Pred}=\frac{0.088}{h}$. Thus, the corrected dietary intake calculation is:

14 $${m}_{Dietary,Metabolit{e}_{i}}={m}_{Dietary}\,\ast \,\chi \,\ast \,{p}_{Metabolit{e}_{i}}$$

where ${m}_{Dietary,Metabolit{e}_{i}}$ is the dietary intake of metabolite *i* in $\frac{g}{h}$, ${m}_{Dietary}$ is the dietary intake in $\frac{g}{h}$, *χ* is the correction factor, and ${p}_{Metabolit{e}_{i}}$ is the proportion of each metabolite *i* in the holidic medium.

For the network modelling, all dietary internalizations have to be flux rates or uptake rates, which we calculated as follows:

15 $${v}_{uptake,i}=\frac{{m}_{Dietary,Metabolit{e}_{i}}}{{M}_{Metabolit{e}_{i}}\,\ast \,{m}_{\Delta ,dry}}$$

where ${v}_{uptake,i}$ is the uptake rate of metabolite *i* in $\frac{mmol}{g\,\ast \,h}$, ${m}_{Dietary,Metabolit{e}_{i}}$ is the dietary intake of metabolite *i* in $\frac{g}{h}$, ${M}_{Metabolit{e}_{i}}$ is the molar mass of metabolite *i* in $\frac{g}{mmol}$, and ${m}_{\Delta ,dry}$ is the difference of dry weight between 168 h and 96 h in *gram*.

### Summary statement

FlySilico, a flux balance analysis suitable metabolic network of *Drosophila melanogaster* is presented, and its use for the investigation of larval growth and metabolism is demonstrated.

## Supplementary information

Supplemental Information

Dataset 1

Dataset 2

Dataset 3

Dataset 4

Dataset 5

## Data Availability

The supplementary material consists of the metabolic network reconstruction (Table S1), the raw data of all measurements (Table S2), the result of the flux variability analysis for the different diet simulations (Table S3) and the corresponding flux changes in relation to the standard HD (Table S4). Additionally, we added interactive versions of the metabolic network map (Interactive Supplementary Fig. 1) and the GAM/NGAM plot (Interactive Supplementary Fig. 2), which corresponds Fig. S6. Further, we provide all raw data, the custom python scripts used for the calculation of the weights of the biomass function and the uptake rates, the model comparison and the main scripts for the model reconstruction and analysis in a zip file for use with the Anaconda Project function (https://anaconda-project.readthedocs.io/en/latest/). Once unzipped, the folder contains all information to create an Anaconda Python environment with the required packages in the appropriate version to run our code and all necessary information to rerun or modify our analyses. A readme file within the folder should guide users through the procedure to get the environment operational. We additionally provide all scripts via GitLab: https://gitlab.com/Beller-Lab/flysilico.
