# Supplementary material for: FlySilico: Flux balance modeling of Drosophila larval growth and resource allocation
Source: Sci Rep. 2019 Nov 20;9:17156. doi: 10.1038/s41598-019-53532-4 (PMC6868164; doi:10.1038/s41598-019-53532-4)
Supplement: Supplementary file 1 — Supplemental Information [file 41598_2019_53532_MOESM1_ESM.pdf]

Supplemental Information File

FlySilico: Flux balance modeling of *Drosophila* larval growth and resource allocation

Jürgen Wilhelm Schönborn, Lisa Jehrke, Tabea Mettler-Altmann, Mathias Beller

We provide all primary data as well as the metabolic network in supplemental tables (please see below). The Python scripts for the flux balance modeling and parameter estimations are provided via GitLab (https://gitlab.com/Beller-Lab/flysilico/) and an Anaconda Project folder to allow reproducible research thanks to a snapshot of a Python working environment with the adequate package versions.

The supplemental information comprises:

- 9 figures (found below)
- Four Supplemental Tables:
    - Table S1: Information for the *Drosophila* metabolic network „FlySilico“
    - Table S2: Wet lab experimental data file
    - Table S3: Flux variability analysis results
    - Table S4: Normalized flux variability results
- Two Supplementary Interactive figures:
    - Supplementary Interactive Figure 1: Interactive Metabolic Network Map
    - Supplementary Interactive Figure 2: Interactive GAM/NGAM/Growth rate plot
- Supplemental Zip File with the code and data necessary to use the FlySilico metabolic network for flux balance analyses and to generate the plots shown in the figures

A

MNXM18_bm + MNXM876_bm + bigg_ala_L_bm + bigg_amp_bm + bigg_arg_L_bm + bigg_asn_L_bm + bigg_asp_L_bm + bigg_atp_bm + bigg_cmp_bm + bigg_cys_L_bm + bigg_damp_bm + bigg_dcmp_bm + bigg_dgmp_bm + bigg_dtmp_bm + bigg_gln_L_bm + bigg_gly_bm + bigg_gmp_bm + bigg_his_L_bm + bigg_ile_L_bm + bigg_leu_L_bm + bigg_lys_L_bm + bigg_met_L_bm + bigg_phe_L_bm + bigg_pro_L_bm + bigg_ser_L_bm + bigg_thr_L_bm + bigg_trp_L_bm + bigg_tyr_L_bm + bigg_ump_bm + bigg_val_L_bm --> bigg_adp_bm + bigg_pi_bm

B

IN FLUXES	OUT FLUXES						OBJECTIVES	
	MNXM10815_i	1e+03	bigg_arachd_i	1e+03	bigg_aicar_i	500	BIOMASS_REAC...	500
	MNXM1158_i	1e+03	bigg_but_i	1e+03	MNXM4091_i	421		
	MNXM12528_i	1e+03	bigg_co2_i	1e+03	MNXM977_i	421		
	MNXM2426_i	1e+03	bigg_co_i	1e+03	bigg_n2_i	411		
	MNXM305_i	1e+03	bigg_cysam_i	1e+03	MNXM59_i	346		
	MNXM369_i	1e+03	bigg_dtdpglu_i	1e+03	bigg_gam1p_i	346		
	MNXM5127_i	1e+03	bigg_gcald_i	1e+03	bigg_maltttr_i	307		
	MNXM53135_i	1e+03	bigg_glyc_i	1e+03	bigg_ins_i	230		
	MNXM7206_i	1e+03	bigg_h2_i	1e+03	MNXM56_i	210		
	MNXM7559_i	1e+03	bigg_no3_i	1e+03	bigg_ascb_L_i	193		
	MNXM7713_i	1e+03	bigg_uamag_i	1e+03	MNXM96041_i	154		
	MNXM92184_i	1e+03	bigg_no2_i	849	bigg_meoh_i	123		
	MNXM9857_i	1e+03	bigg_hpyr_i	689	MNXM18606_i	116		
	bigg_5aop_i	1e+03	MNXM1128_i	579	MNXM1289_i	67.2		
	bigg_ade_i	1e+03	MNXM539_i	500	bigg_alaala_i	39.4		

Figure S1: Modeling growth with the computer generated *Drosophila* metabolic network (BMID000000141998; https://www.ebi.ac.uk/biomodels-main/BMID000000141998). (A) Computational biomass function as provided from the ebi website. (B) FBA solution with no substrate incorporation (missing “in fluxes”). The model predicts a biomass production (column “objectives” shows a value of 500 for the biomass reaction). bm – biomass | i - intra cellular | MNXM18_bm - glutamate(1-) | MNXM876_bm - glycogen | bigg_ala_L_bm - L-alanine zwitterion | bigg_amp_bm - AMP | bigg_arg_L_bm - L-argininium(1+) | bigg_asn_L_bm - L-asparagine zwitterion | bigg_asp_L_bm - L-aspartate(1-) | bigg_atp_bm - ATP | bigg_cmp_bm - CMP | bigg_cys_L_bm - L-cysteine zwitterion | bigg_damp_bm - dAMP | bigg_dcmp_bm - dCMP(2-) | bigg_dgmp_bm - dGMP(2-) | bigg_dtmp_bm - dTMP | bigg_gln_L_bm - L-glutamine zwitterion | bigg_gly_bm - glycine | bigg_gmp_bm - GMP(3-) | bigg_his_L_bm - L-histidine zwitterion | bigg_ile_L_bm - L-isoleucine zwitterion | bigg_leu_L_bm - L-leucine zwitterion | bigg_lys_L_bm - L-lysinium(1+) | bigg_met_L_bm - L-methionine zwitterion | bigg_phe_L_bm - L-phenylalanine | bigg_pro_L_bm - L-proline | bigg_ser_L_bm - L-serine | bigg_thr_L_bm - L-threonine zwitterion | bigg_trp_L_bm - L-tryptophan zwitterion | bigg_tyr_L_bm - L-tyrosine zwitterion | bigg_ump_bm - UMP(2-) | bigg_val_L_bm - L-valine zwitterion | bigg_adp_bm - ADP | bigg_pi_bm - phosphate | MNXM10815_i - beta-methylenecyclopropyl pyruvate | MNXM1158_i - sn-glycerol 1-phosphate(2-) | MNXM12528_i - an \xc5\x93\xc3\xa2-oxo fatty acid | MNXM2426_i - ribonucleoside | MNXM305_i - 2-methyl-3-oxopropanoate | MNXM369_i - 1L-myo-inositol 1,2,3,4,6-pentakisphosphate(10-) | MNXM5127_i - diethylphosphate | MNXM53135_i - fatty aldehyde | MNXM7206_i - a debranched limit dextrin | MNXM7559_i - N-acetyl-D-galactosaminyl-polypeptide | MNXM7713_i - a [protein]-L-tyrosine | MNXM92184_i - CMP-N-glycoloylneuraminate | MNXM9857_i - 2-methylamine-furan phosphate | bigg_5aop_i - 5-aminolevulinate | bigg_ade_i - adenine | bigg_arachd_i - arachidonate | bigg_but_i - butyrate | bigg_co2_i - CO(2) | bigg_co_i - carbon monoxide(1+) | bigg_cysam_i - cysteaminium | bigg_dtdpglu_i - dTDP-alpha-D-glucose | bigg_gcald_i - glycolaldehyde | bigg_glyc_i - alditol | bigg_h2_i - H2 | bigg_no3_i - nitrate | bigg_uamag_i - UDP-N-acetylmuramoyl-L-alanyl-D-glutamate(4-) | bigg_no2_i - nitrite | bigg_hpyr_i - 3-hydroxypyruvate | MNXM1128_i - 1-aminocyclopropanecarboxylic acid zwitterion | MNXM539_i - 1-acyl-sn-glycero-3-phosphoglycerol | bigg_aicar_i - 5-amino-1-(5-phospho-D-ribosyl)imidazole-4-carboxamide(2-) | MNXM4091_i - psoralen | MNXM977_i - ethene | bigg_n2_i - dinitrogen | MNXM59_i - 1,2-diacyl-sn-glycerol | bigg_gam1p_i - alpha-D-glucosamine 1-phosphate(1-) | bigg_maltttr_i - alpha-maltotetraose | bigg_ins_i - inosine | MNXM56_i - formaldehyde | bigg_ascb_L_i - ascorbate | MNXM96041_i - 1-acylglycerophosphocholine | bigg_meoh_i - methanol | MNXM18606_i - hyperforin | MNXM1289_i - oxalatosuccinate(3-) | bigg_alaala_i - D-alanyl-D-alanine

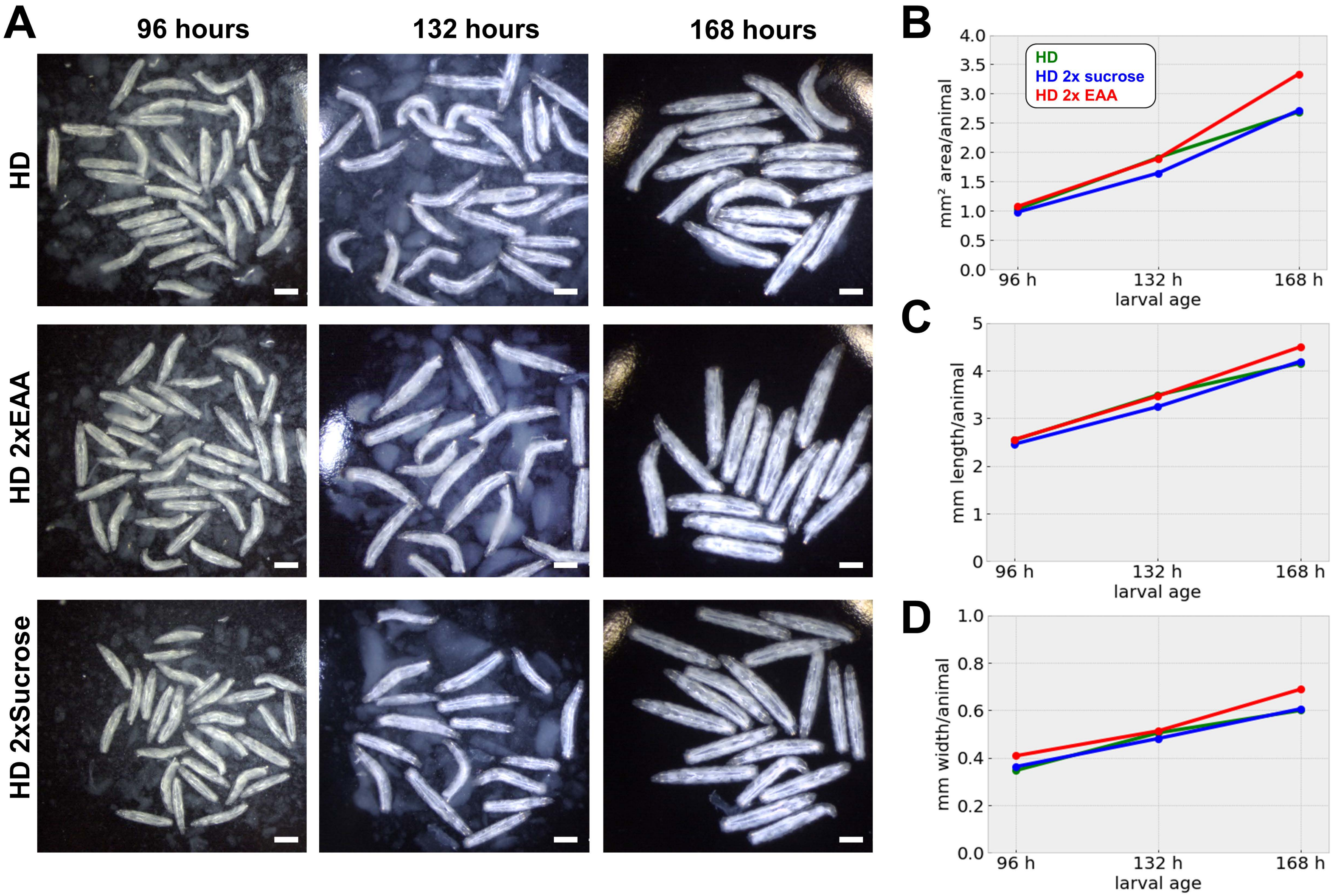
A
96 hours
132 hours
168 hours
HD
HD 2xEAA
HD 2xSucrose
B
HD
HD 2x sucrose
HD 2x EAA
mm² area/animal
larval age
C
mm length/animal
larval age
D
mm width/animal
larval age

Figure S2: Larval size measurements. (A) Larvae were raised on HD, HD with 2x sucrose or HD with 2x EAA and images were recorded at the indicated time points under a dissecting microscope. The images served the subsequent quantification of the area (B), length (C) and width (D) of the animals across development. Color code in (B-D) is: HD = green, HD with 2x sucrose = blue, and HD with 2x EAA = red. Scale bars in (A) represent 1 mm.

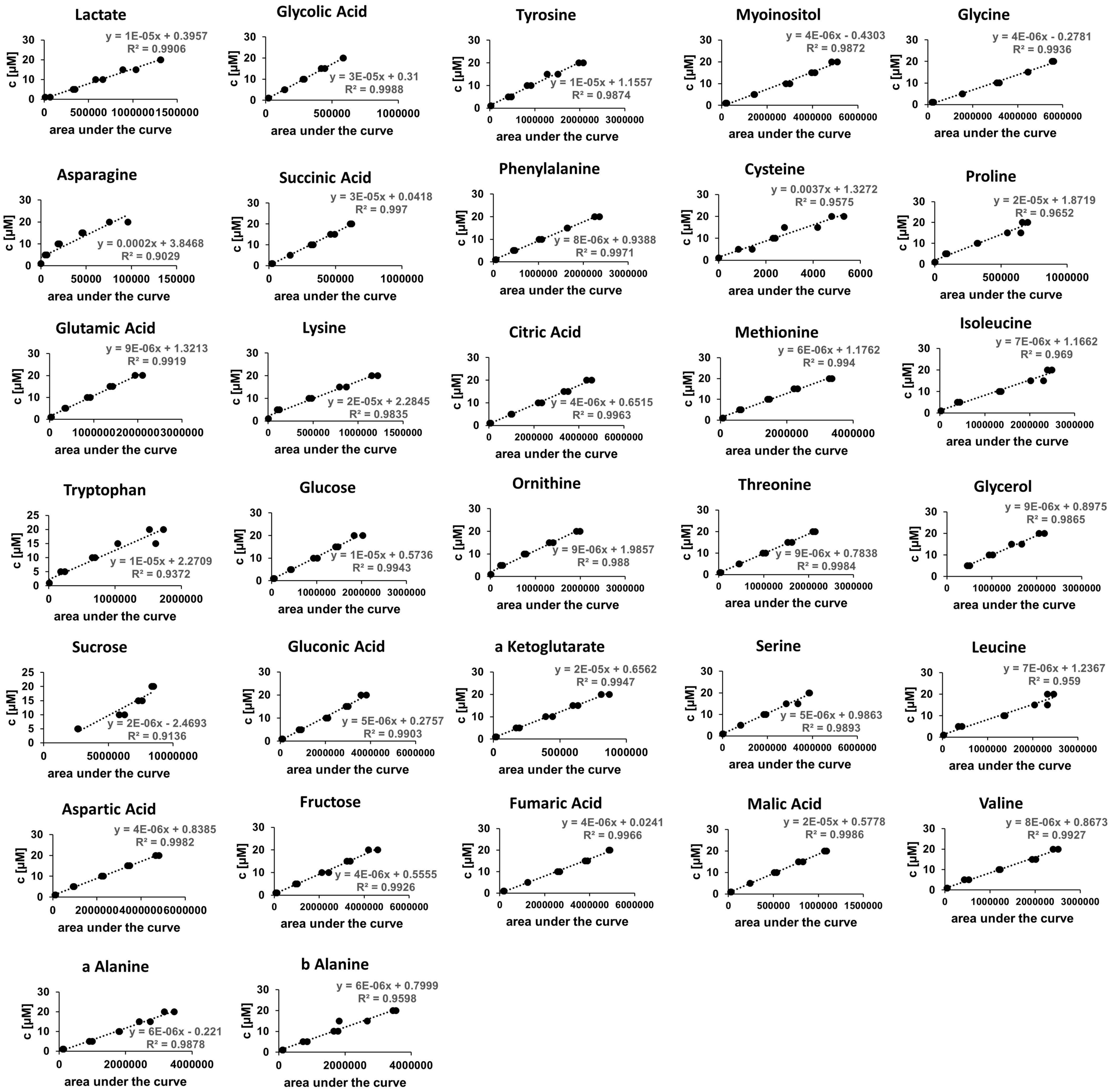
c [µM]
area under the curve
Lactate
y = 1E-05x + 0.3957
R² = 0.9906
Glycolic Acid
y = 3E-05x + 0.31
R² = 0.9988
Tyrosine
y = 1E-05x + 1.1557
R² = 0.9874
Myoinositol
y = 4E-06x - 0.4303
R² = 0.9872
Glycine
y = 4E-06x - 0.2781
R² = 0.9936
Asparagine
y = 0.0002x + 3.8468
R² = 0.9029
Succinic Acid
y = 3E-05x + 0.0418
R² = 0.997
Phenylalanine
y = 8E-06x + 0.9388
R² = 0.9971
Cysteine
y = 0.0037x + 1.3272
R² = 0.9575
Proline
y = 2E-05x + 1.8719
R² = 0.9652
Glutamic Acid
y = 9E-06x + 1.3213
R² = 0.9919
Lysine
y = 2E-05x + 2.2845
R² = 0.9835
Citric Acid
y = 4E-06x + 0.6515
R² = 0.9963
Methionine
y = 6E-06x + 1.1762
R² = 0.994
Isoleucine
y = 7E-06x + 1.1662
R² = 0.969
Tryptophan
y = 1E-05x + 2.2709
R² = 0.9372
Glucose
y = 1E-05x + 0.5736
R² = 0.9943
Ornithine
y = 9E-06x + 1.9857
R² = 0.988
Threonine
y = 9E-06x + 0.7838
R² = 0.9984
Glycerol
y = 9E-06x + 0.8975
R² = 0.9865
Sucrose
y = 2E-06x - 2.4693
R² = 0.9136
Gluconic Acid
y = 5E-06x + 0.2757
R² = 0.9903
a Ketoglutarate
y = 2E-05x + 0.6562
R² = 0.9947
Serine
y = 5E-06x + 0.9863
R² = 0.9893
Leucine
y = 7E-06x + 1.2367
R² = 0.959
Aspartic Acid
y = 4E-06x + 0.8385
R² = 0.9982
Fructose
y = 4E-06x + 0.5555
R² = 0.9926
Fumaric Acid
y = 4E-06x + 0.0241
R² = 0.9966
Malic Acid
y = 2E-05x + 0.5778
R² = 0.9986
Valine
y = 8E-06x + 0.8673
R² = 0.9927
a Alanine
y = 6E-06x - 0.221
R² = 0.9878
b Alanine
y = 6E-06x + 0.7999
R² = 0.9598

Figure S3: GC-MS calibration curves. Five-point calibration curves for the GC-MS metabolomics measurements (data provided in Table S2).

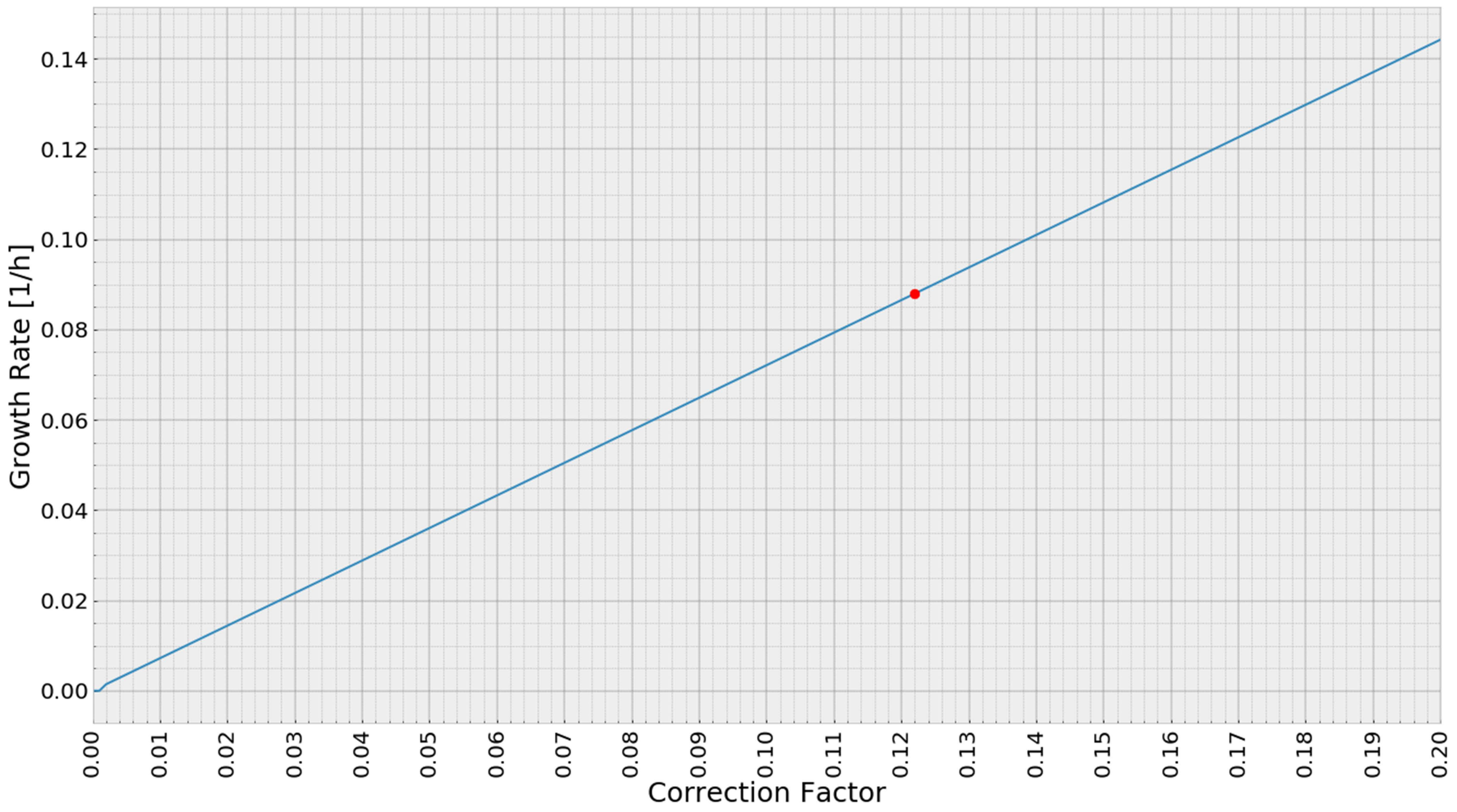
Growth Rate [1/h]
0.14
0.12
0.10
0.08
0.06
0.04
0.02
0.00
0.00
0.01
0.02
0.03
0.04
0.05
0.06
0.07
0.08
0.09
0.10
0.11
0.12
0.13
0.14
0.15
0.16
0.17
0.18
0.19
0.20
Correction Factor

Figure S4: Larval food intake correction factor. Determination of a correction factor for the calculation of the larval food intake rate (for details see main text and methods section).

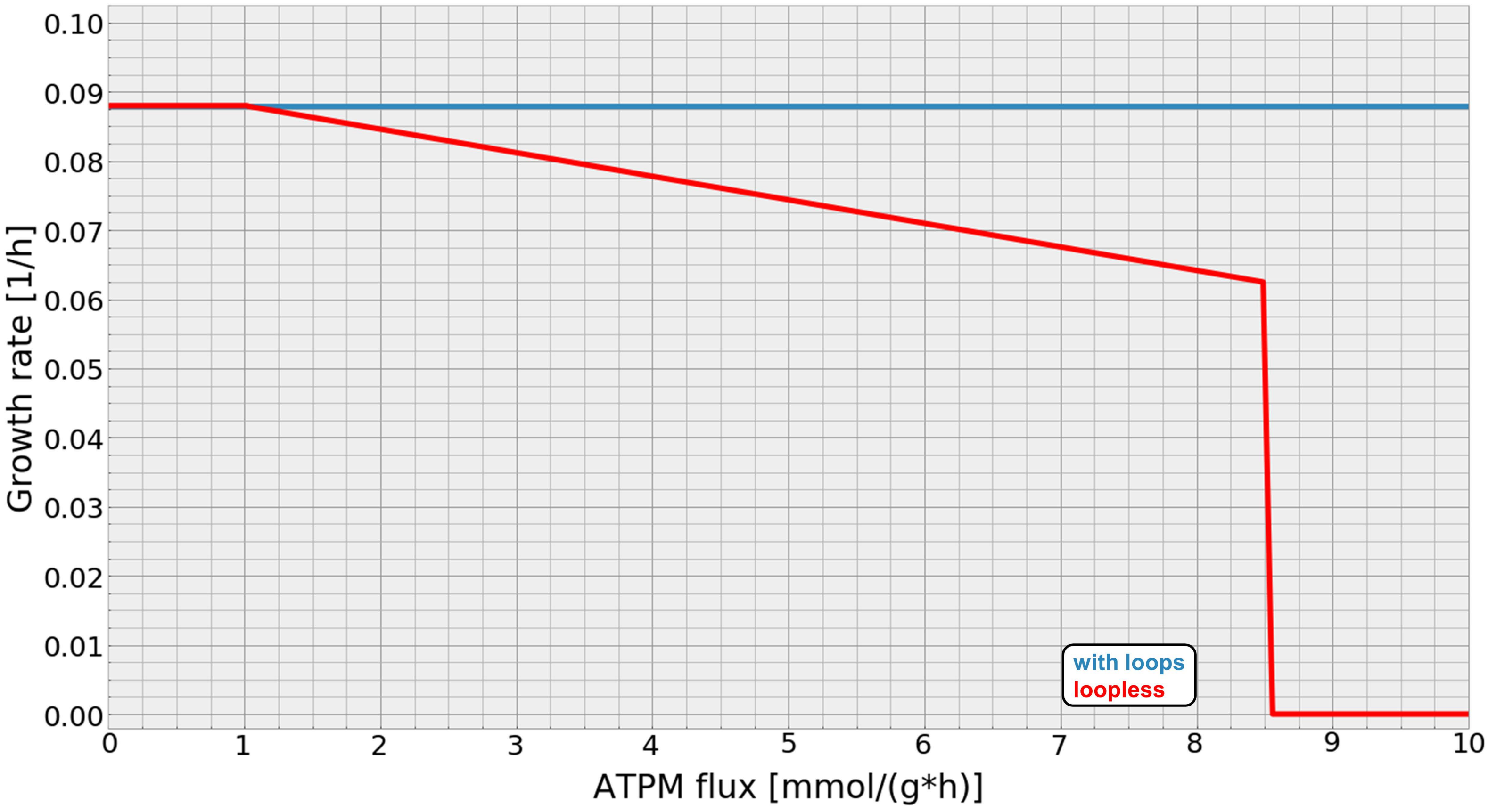

Growth rate [1/h]
0.10
0.09
0.08
0.07
0.06
0.05
0.04
0.03
0.02
0.01
0.00
0
1
2
3
4
5
6
7
8
9
10
ATPM flux [mmol/(g*h)]
with loops
loopless

Figure S5: NGAM value determination. Iterative determination of the NGAM value by fixing the oxygen uptake rate to the oxygen consumption rate of S2R+ cells $\left(0.4788\ \frac{mmol}{g\ dry\ weight*h}\right)$. The last ATPM flux value where growth was still possible was used as the NGAM value $\left(8.55\ \frac{mmol}{g\ dry\ weight*h}\right)$.

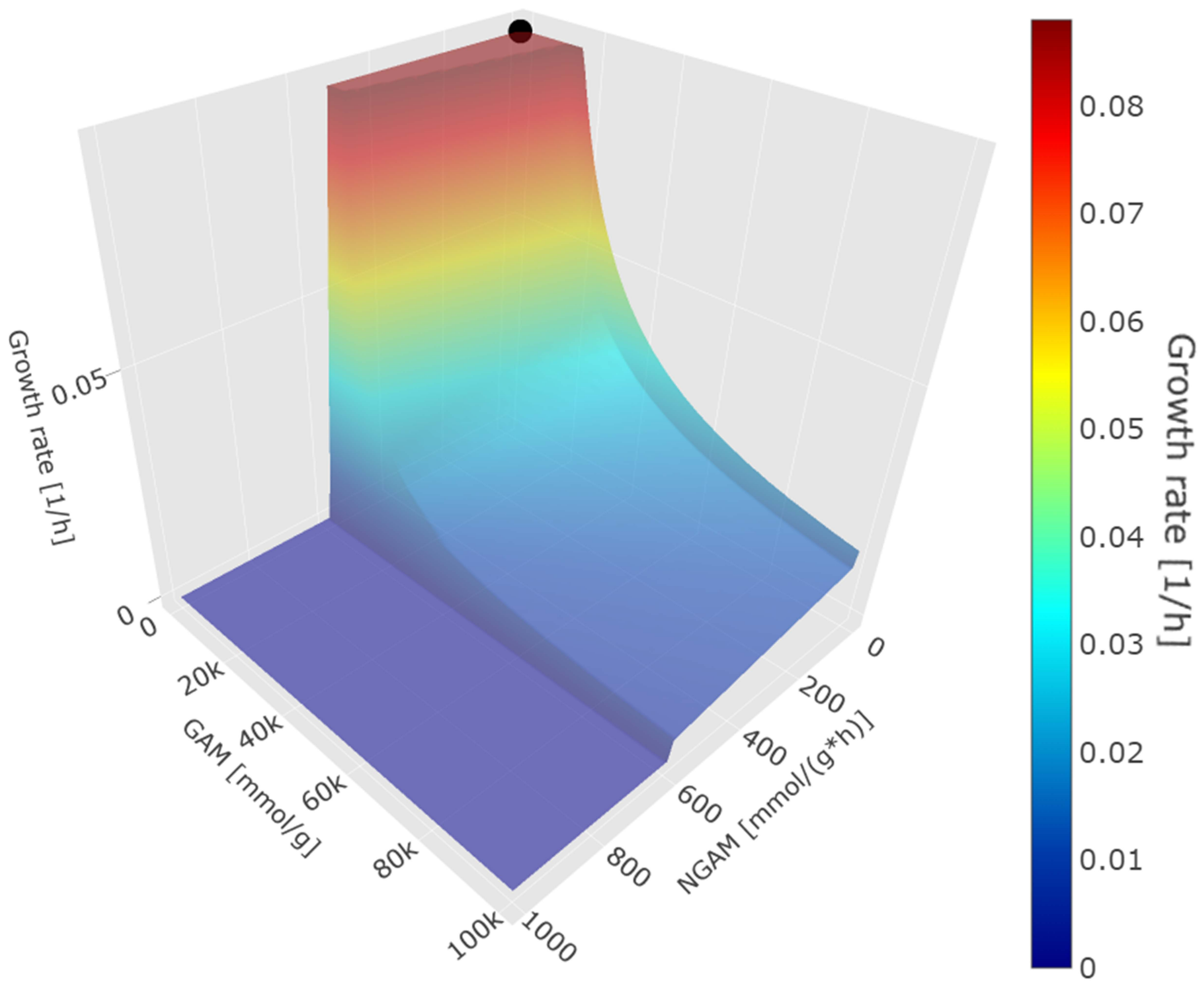
Growth rate [1/h]
0.05
0
0
20k
40k
60k
80k
100k
GAM [mmol/g]
0
200
400
600
800
1000
NGAM [mmol/(g*h)]
0.08
0.07
0.06
0.05
0.04
0.03
0.02
0.01
0
Growth rate [1/h]

Figure S6: Impact of different GAM and NGAM coefficients on growth rate. Over a large range of values, GAM and NGAM do not prominently affect the maximal growth rate. Only very large values for GAM and NGAM result in a decreased growth rate or the complete cessation of biomass production. The black dot indicates the value set used for NGAM and GAM in our simulations. An interactive version of the figure is provided as Interactive Supplementary Figure 2.

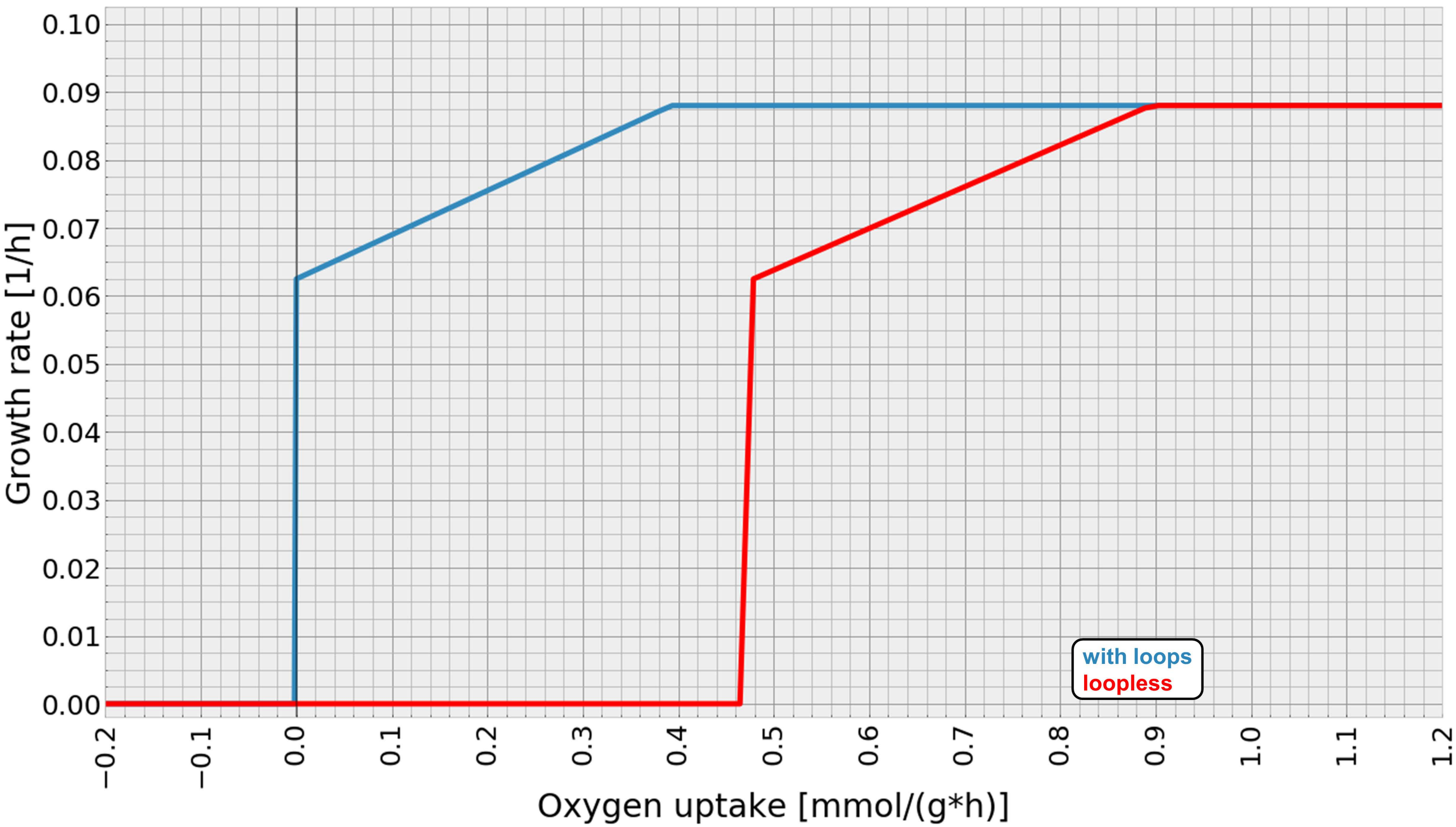

0.10
0.09
0.08
0.07
0.06
0.05
0.04
0.03
0.02
0.01
0.00
Growth rate [1/h]
−0.2
−0.1
0.0
0.1
0.2
0.3
0.4
0.5
0.6
0.7
0.8
0.9
1.0
1.1
1.2
Oxygen uptake [mmol/(g*h)]
with loops
loopless

Figure S7: Growth rate solution comparison of loopless and with loop computations. Comparison between solving the FBA with loops (blue color) and loopless (red color). The solution with loops allows growth in the absence of oxygen, which is a biologically infeasible solution. All modeling steps were thus performed using loopless computations.

Amino acid	Frequency f_{AA}
Alanine	0.074
Arginine	0.055
Asparagine	0.047
Aspartic acid	0.052
Cysteine	0.020
Glutamic acid	0.066
Glutamine	0.054
Glycine	0.062
Histidine	0.026
Isoleucine	0.048
Leucine	0.087
Lysine	0.055
Methionine	0.022
Phenylalanine	0.033
Proline	0.058
Serine	0.086
Threonine	0.059
Tryptophan	0.009
Tyrosine	0.028
Valine	0.059

Figure S8: Frequency of amino acids in the *Drosophila* proteome. As we were missing amino acid measurements in the GC-MS/MS experiments, we sought to identify the remaining coefficients by bioinformatics. For details, see methods section.

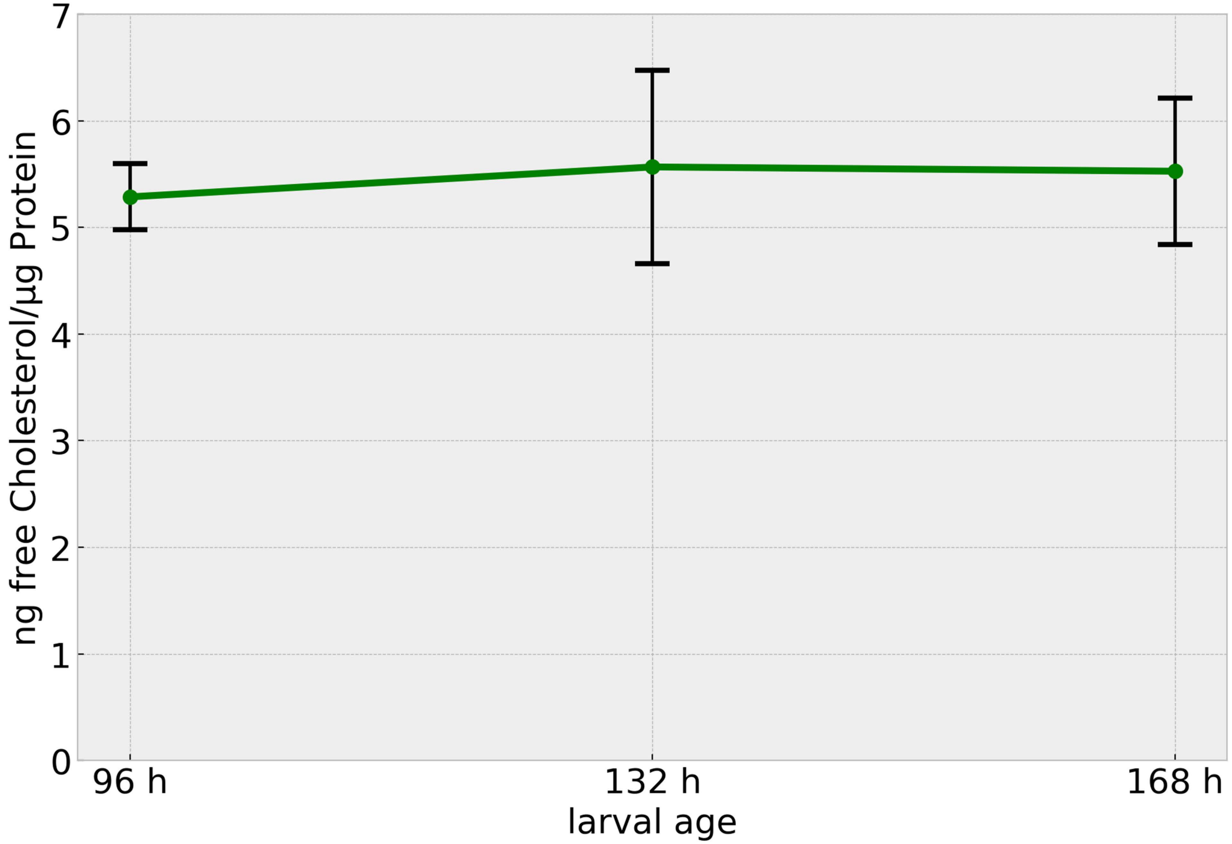

7
6
5
4
3
2
1
0
ng free Cholesterol/µg Protein
96 h
132 h
168 h
larval age

Figure S9: **Free cholesterol measurements.** We quantified the cholesterol content of larvae reared on the holidic diet (between 96 h and 168 h after egg laying). The determined levels were almost constant.

Interactive Supplementary Figure 1: Interactive version of the FlySilico metabolic network (relates to Fig. 2). For download of the figure, please follow the link to the GitLab repository of the project:

https://gitlab.com/Beller-Lab/flysilico/tree/master/Supplementary_Data

The downloaded html figure file opens in the standard web browser of the system. Please mouse over the different elements of the network to obtain more information, as e.g. the nodes (metabolite details), the edges (reaction details) or the dots in the upper right corners of the colored boxes (reaction block details).

Interactive Supplementary Figure 2 (relates to Fig. S6): Interactive version of the GAM / NGAM simulation plot. For download of the figure, please follow the link to the GitLab repository of the project:

https://gitlab.com/Beller-Lab/flysilico/tree/master/Supplementary_Data

The downloaded html figure file opens in the standard web browser of the system. It can be rotated in all three axis and zoomed / repositioned with the mouse pointer.
