# Supplementary material for: FlySilico: Flux balance modeling of Drosophila larval growth and resource allocation
Source: Sci Rep. 2019 Nov 20;9:17156. doi: 10.1038/s41598-019-53532-4 (PMC6868164; doi:10.1038/s41598-019-53532-4)
Supplement: Supplementary file 6 — Dataset 5 [file 41598_2019_53532_MOESM6_ESM.zip › FlySilico/Comparison/Model_comparison.docx]

Table 3: Model comparison between different organisms. . * Same value in non-loopless and loopless. ** impossible to compute for loopless model in time.

| Organism | Model ID | Reactions | Metabolites | Compartments | Number of dead-end  reactions | Number of blocked  reactions | Number of unbalanced  reactions | Number of exchange  reactions |
| --- | --- | --- | --- | --- | --- | --- | --- | --- |
| *Escherichia coli* | iWFL_1372  (Monk et al. 2013) | 2782 | 1973 | pp, c, e | 264 (9.5%)* | 379 (13.6%) / 385 (13.8%) | 39 (1.4%)* | 391 (14.1%)* |
| *Escherichia coli* | e_coli_core  (Orth et al. 2010a) | 95 | 72 | c, e | 0 (0.0%)* | 0 (0.0%)* | 1 (1.1%)* | 20 (21.1%)* |
| *Homo sapiens* | RECON1  (Duarte et al. 2007) | 3741 | 2766 | c, e, g, m, l, n, er, p | 434 (11.6%)* | 1274 (34.1%)** | 55 (1.5%) / 56 (1.5%) | 430 (11.5%)* |
| *Homo sapiens* | Recon3D (Brunk et al. 2018) | 10600 | 5835 | c, e, g, m, l, n, er, p | 0 (0.0%)* | 0 (0.0%) ** | 3077 (29.0%) /  3076 (29.0%) | 1806 (17.0%)* |
| *Mus musculus* | iMM1415 (Sigurdsson et al. 2010) | 3726 | 2775 | c, e, g, m, l, n, er, p | 518 (13.9%)* | 792 (21.3%)** | 0 (0.0%)* | 454 (12.2%)* |
| *Saccharomyces cerevisiae* | iMM904 (Mo et al. 2009) | 1577 | 1226 | c, e, g, m, v, n, er, p | 213 (13.5%)* | 553 (35.1%) / 543 (34.4%) | 51 (3.2%) / 48 (3.0%) | 164 (10.4%)* |
| *Drosophila melanogaster* | FlySilico_v1 | 363 | 293 | c, e, m | 9 (2.5%)* | 10 (2.8%) /  13 (3.6%) | 2 (0.6%)* | 64 (17.6%)* |
| *Drosophila melanogaster* | BMID000000141998 (EMBL-EBI) | 6198 | 2873 | e, c | 12 (0.2%)* | 12 (0.2%)** | 407 (6.6%)* | 2865 (46.2%)* |
