# Supplementary material for: FlySilico: Flux balance modeling of Drosophila larval growth and resource allocation
Source: Sci Rep. 2019 Nov 20;9:17156. doi: 10.1038/s41598-019-53532-4 (PMC6868164; doi:10.1038/s41598-019-53532-4)
Supplement: Supplementary file 6 — Dataset 5 [file 41598_2019_53532_MOESM6_ESM.zip › FlySilico/Experiments/experiment01_diff-diets/V50vsV46.pptx]

## Slide 1
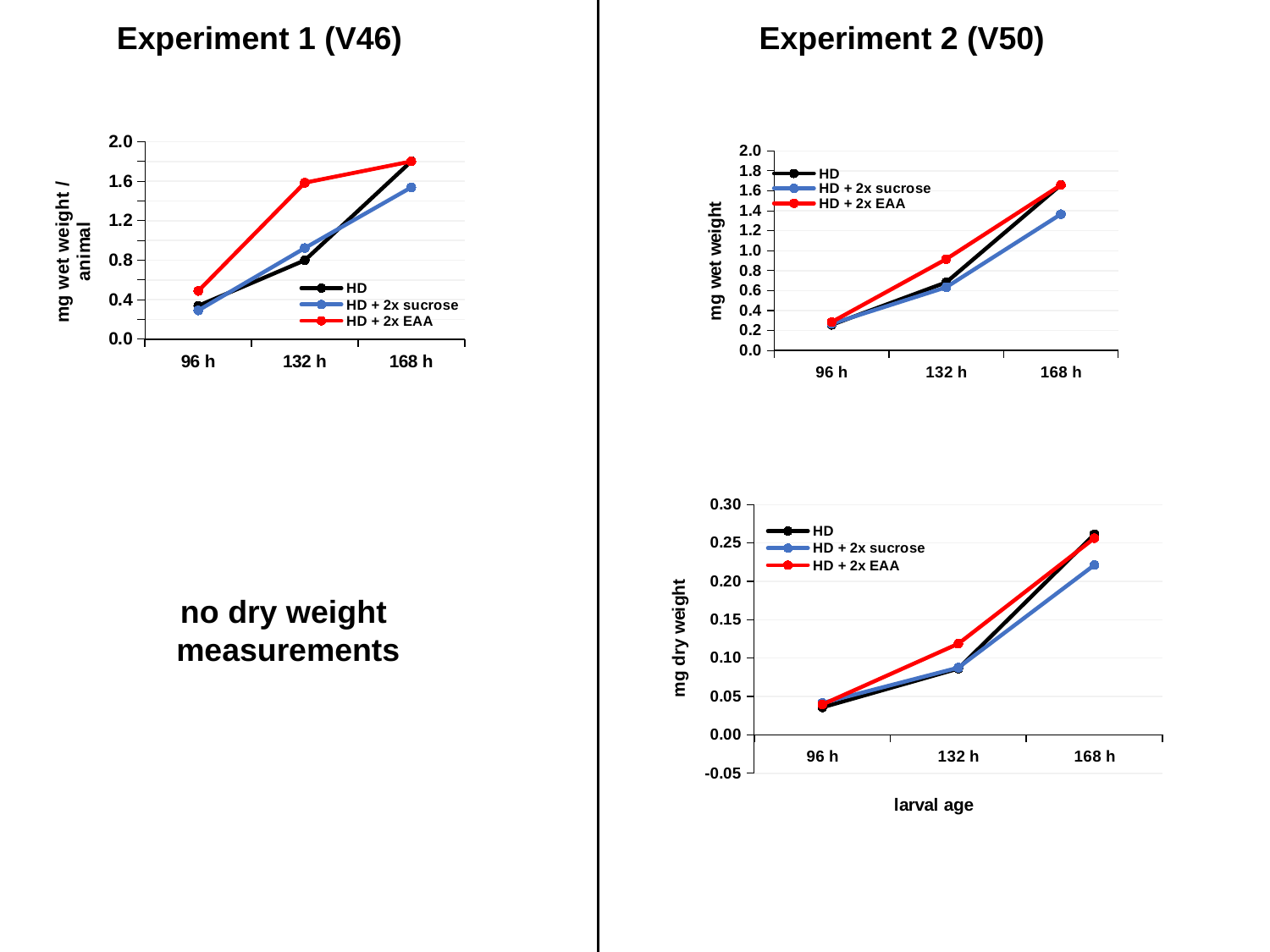

Experiment 1 (V46)
Experiment 2 (V50)
### Chart
| Category | | | |
|---|---|---|---|
| 96 h | 0.3381250000000002 | 0.291312499999998 | 0.48799999999999955 |
| 132 h | 0.7986805555555564 | 0.9227083333333332 | 1.584374999999999 |
| 168 h | 1.801041666666671 | 1.5379166666666677 | 1.800000000000003 |
### Chart
| Category | | | |
|---|---|---|---|
| 96 h | 0.2567500000000006 | 0.26725 | 0.28400000000000036 |
| 132 h | 0.6824999999999998 | 0.633125000000001 | 0.9156250000000004 |
| 168 h | 1.659375000000002 | 1.3656250000000008 | 1.6575000000000006 |
### Chart
| Category | | | |
|---|---|---|---|
| 96 h | 0.035750000000000504 | 0.04149999999999987 | 0.03950000000000009 |
| 132 h | 0.0862500000000016 | 0.08750000000000147 | 0.1187500000000008 |
| 168 h | 0.26125000000000176 | 0.2212500000000006 | 0.2562500000000009 |no dry weight
measurements

## Slide 2
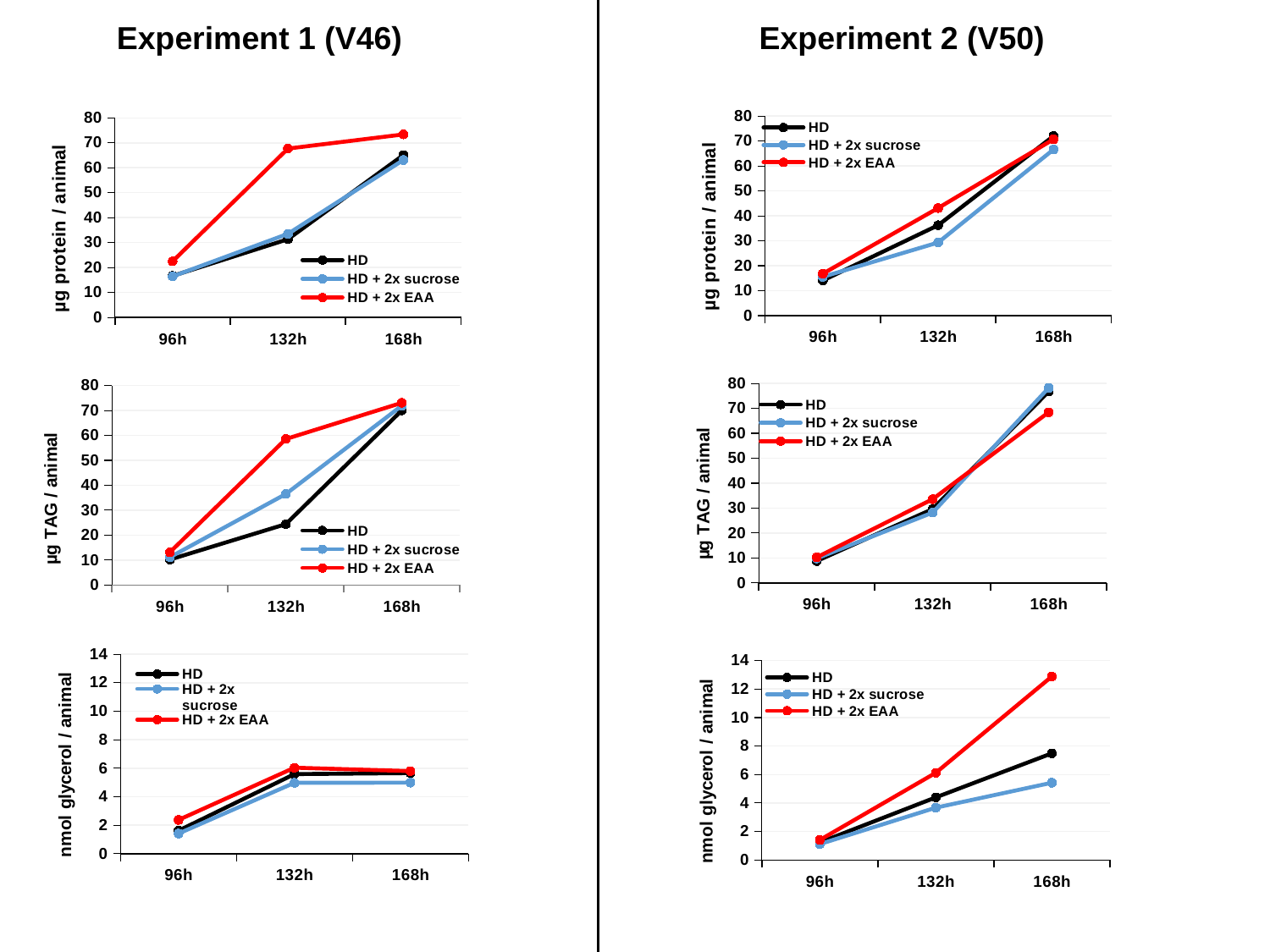

Experiment 1 (V46)
Experiment 2 (V50)
### Chart
| Category | | HD + 2x sucrose | HD + 2x EAA |
|---|---|---|---|
| 96h | 14.075683 | 15.531021000000003 | 16.793599 |
| 132h | 36.245990625000005 | 29.30361875 | 43.143184375 |
| 168h | 71.996946875 | 66.605690625 | 70.68678125 |
### Chart
| Category | HD | HD + 2x sucrose | HD + 2x EAA |
|---|---|---|---|
| 96h | 16.6318446 | 16.4433726 | 22.474476600000006 |
| 132h | 31.330940625000004 | 33.510148125 | 67.700146875 |
| 168h | 65.07920812500001 | 63.106141875000006 | 73.36903125 |
### Chart
| Category | HD | HD + 2x sucrose | HD + 2x EAA |
|---|---|---|---|
| 96h | 8.7697778 | 9.8160268 | 10.274114200000001 |
| 132h | 29.597023125 | 28.200846249999998 | 33.555803125 |
| 168h | 76.71357437500001 | 78.162770625 | 68.40720562500002 |
### Chart
| Category | HD | HD + 2x sucrose | HD + 2x EAA |
|---|---|---|---|
| 96h | 10.234316000000002 | 11.168232 | 13.148584 |
| 132h | 24.422299999999996 | 36.5182 | 58.565087500000004 |
| 168h | 70.0280625 | 71.9268375 | 73.0872 |
### Chart
| Category | HD | HD + 2x sucrose | HD + 2x EAA |
|---|---|---|---|
| 96h | 1.6181500000000002 | 1.4141000000000001 | 2.3709 |
| 132h | 5.584687500000001 | 4.976718750000001 | 6.033906250000001 |
| 168h | 5.656718750000001 | 4.987343750000001 | 5.7946875 |
### Chart
| Category | HD | HD + 2x sucrose | HD + 2x EAA |
|---|---|---|---|
| 96h | 1.24045 | 1.1240999999999999 | 1.4117500000000003 |
| 132h | 4.3959375000000005 | 3.6762499999999996 | 6.120625 |
| 168h | 7.48828125 | 5.425937500000001 | 12.87859375 |

## Slide 3
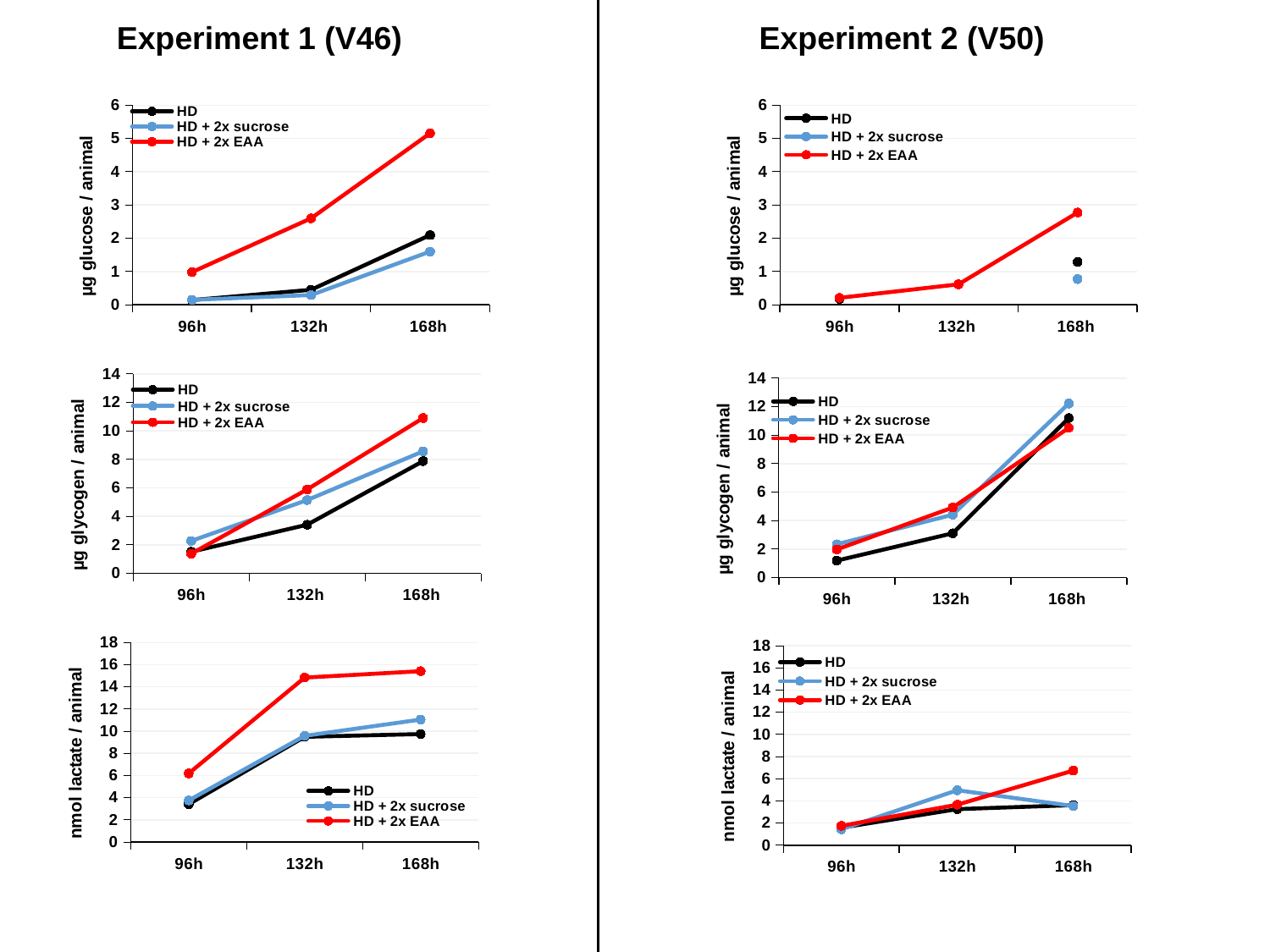

Experiment 1 (V46)
Experiment 2 (V50)
### Chart
| Category | HD | HD + 2x sucrose | HD + 2x EAA |
|---|---|---|---|
| 96h | 0.1382772 | 0.14667297777777777 | 0.9795341333333334 |
| 132h | 0.44785833333333325 | 0.29043749999999974 | 2.5966527083333335 |
| 168h | 2.0929060416666667 | 1.5970304166666667 | 5.154741249999999 |
### Chart
| Category | HD | HD + 2x sucrose | HD + 2x EAA |
|---|---|---|---|
| 96h | 0.17726083333333334 | None | 0.20604833333333333 |
| 132h | None | None | 0.6139140625000001 |
| 168h | 1.2856223958333337 | 0.7698463541666667 | 2.7729765625000002 |
### Chart
| Category | HD | HD + 2x sucrose | HD + 2x EAA |
|---|---|---|---|
| 96h | 1.5207058666666666 | 2.2786732000000005 | 1.3788300666666669 |
| 132h | 3.414575208333334 | 5.145545000000001 | 5.889701041666667 |
| 168h | 7.882068541666667 | 8.567405 | 10.903279375000004 |
### Chart
| Category | HD | HD + 2x sucrose | HD + 2x EAA |
|---|---|---|---|
| 96h | 1.1928017000000002 | 2.3386070333333335 | 1.9740696 |
| 132h | 3.110072604166667 | 4.415091979166667 | 4.917519791666667 |
| 168h | 11.195432708333335 | 12.214348750000001 | 10.509957916666668 |
### Chart
| Category | HD | HD + 2x sucrose | HD + 2x EAA |
|---|---|---|---|
| 96h | 3.4143 | 3.7584199999999996 | 6.19092 |
| 132h | 9.500249999999998 | 9.5838125 | 14.8390625 |
| 168h | 9.7465625 | 11.051625000000001 | 15.416562500000001 |
### Chart
| Category | HD | HD + 2x sucrose | HD + 2x EAA |
|---|---|---|---|
| 96h | 1.59022 | 1.44388 | 1.7499399999999998 |
| 132h | 3.26225 | 4.9624999999999995 | 3.66125 |
| 168h | 3.6224375 | 3.541625 | 6.737562500000001 |
