# Supplementary material for: FlySilico: Flux balance modeling of Drosophila larval growth and resource allocation
Source: Sci Rep. 2019 Nov 20;9:17156. doi: 10.1038/s41598-019-53532-4 (PMC6868164; doi:10.1038/s41598-019-53532-4)
Supplement: Supplementary file 6 — Dataset 5 [file 41598_2019_53532_MOESM6_ESM.zip › FlySilico/readme_1p1.html]

readme\_1p1


# **FlySilico 1.0 Environment creation**¶

###### by Lisa Jehrke, Mathias Beller and Jürgen Schönborn¶

---

### Follow the guide if you want to use FlySilico with a compatible environment with all necessary packages¶

---

## Requirements:¶

### - *Python 2.7*¶

### - *Anaconda* (also works with Miniconda, manually download of anaconda-project is required)¶

### - *anaconda-project* (working version: 0.8.2)¶

### - ***Gurobi*** (Version 8.0 was used)¶

##### IMPORTANT: The calculations are done with a solver capable to calculate MILP-Problems! Solver that cant deal with MILP-Problems are most likely to fall in a infinite loop while calculating without an error! For more info about loopless FBA: **Schellenberger et. al.**

---

# Steps:¶

### 1. Start your Anaconda Prompt that you have installed with Anaconda/Miniconda¶

### 2. Navigate to the extracted FlySilico folder¶

### 3. Run the anaconda-project.yml by executing "anaconda-project run". It will download every required package and creates an environment in your FlySilico folder. Depending on your computer speed and internet connection speed this could take a while (size of the new environment folder is ~1.7 GB).¶

##### IMPORTANT: If you get any error (most likely) ignore it because anaconda-project tries to start a notebook with the current environment and not with the recently downloaded/created environment!

### 4. Activate your new environment by executing "conda activate envs/default"¶

### 5. Now you can start a jupyter notebook to use all provided scripts¶
