# Supplementary figures and images for: FlySilico: Flux balance modeling of Drosophila larval growth and resource allocation
Source: Sci Rep. 2019 Nov 20;9:17156. doi: 10.1038/s41598-019-53532-4 (PMC6868164; doi:10.1038/s41598-019-53532-4)

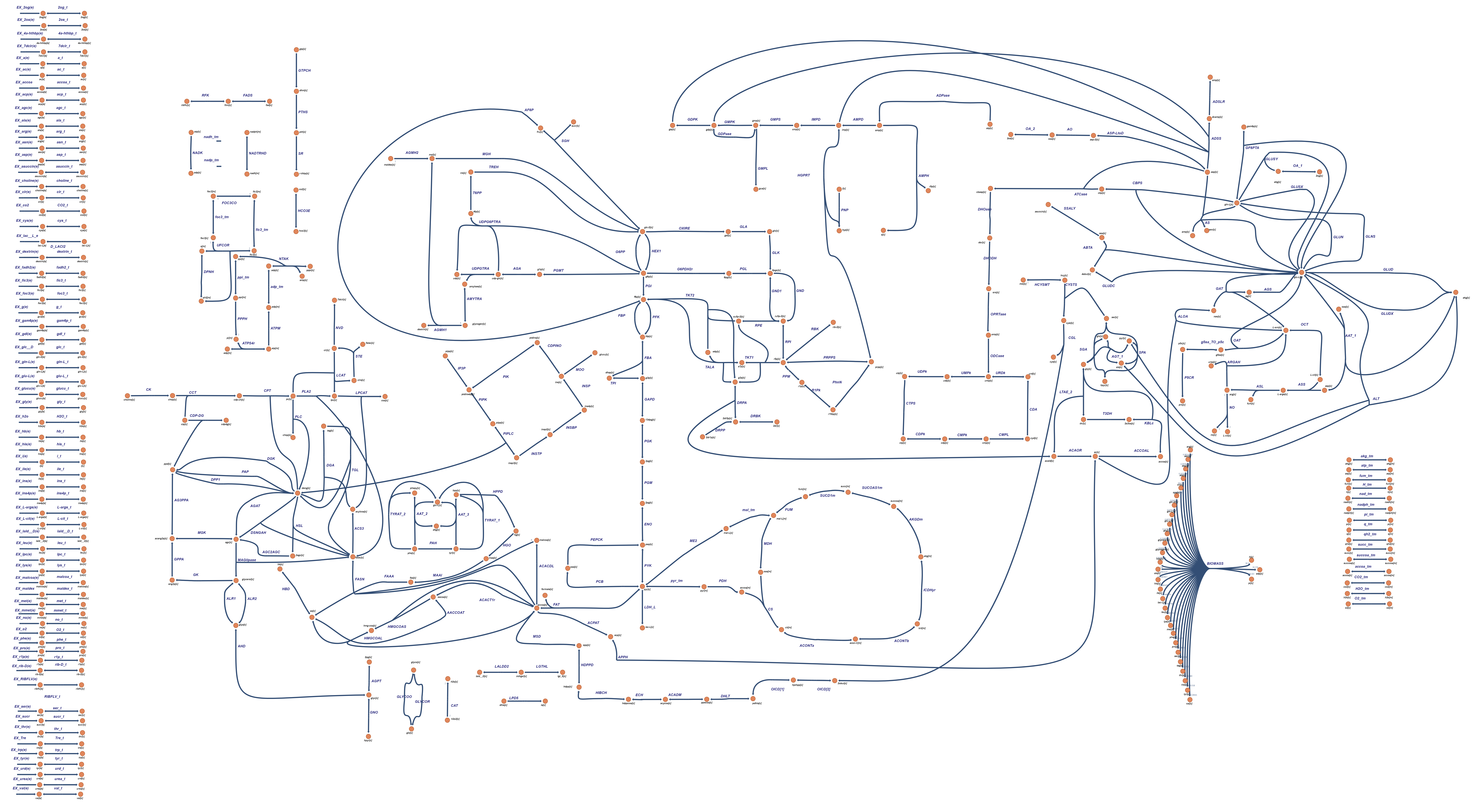

Supplement: Supplementary file 6 — Dataset 5 [file 41598_2019_53532_MOESM6_ESM.zip › FlySilico/flysilico_background.png]

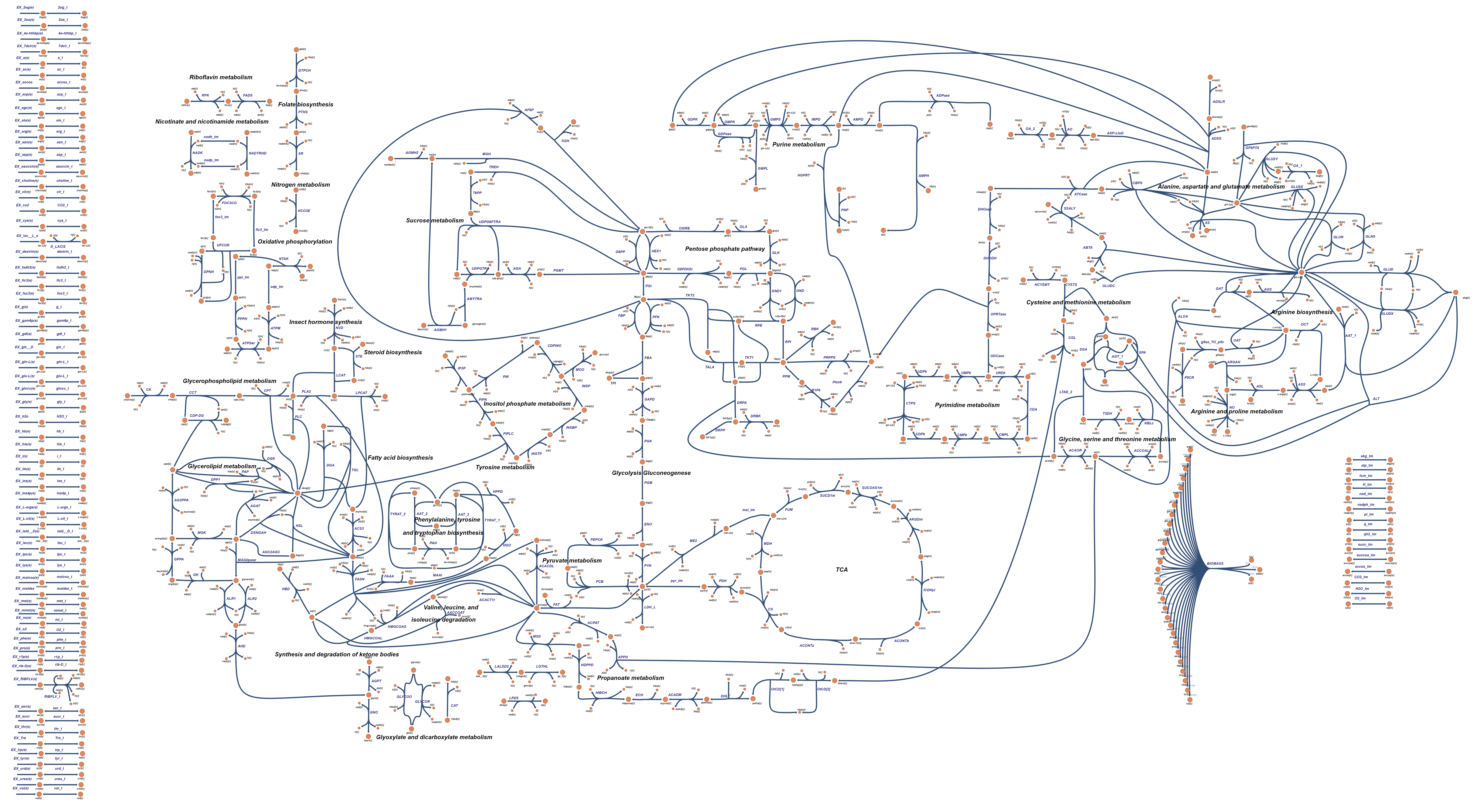

Supplement: Supplementary file 6 — Dataset 5 [file 41598_2019_53532_MOESM6_ESM.zip › FlySilico/flysilico_v1_map.png]

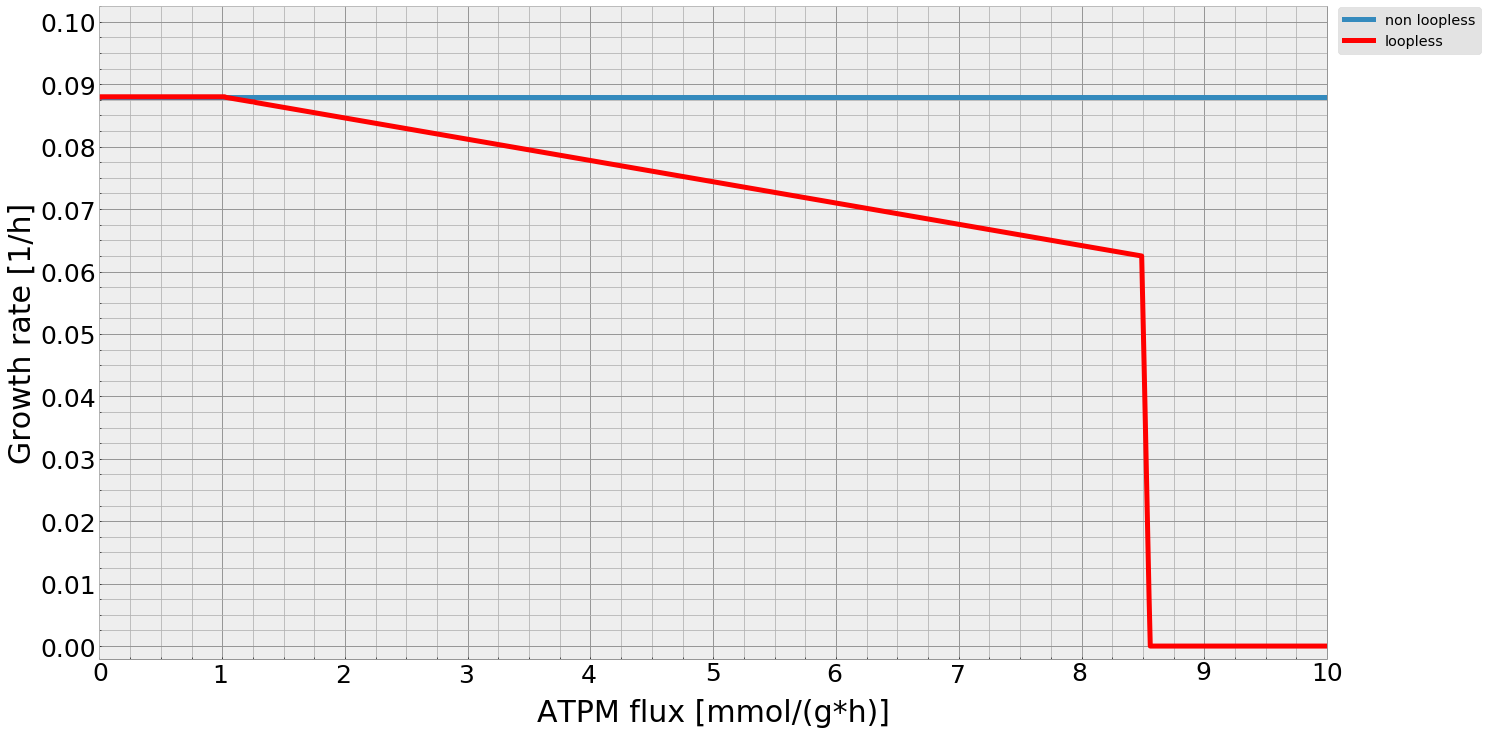

Supplement: Supplementary file 6 — Dataset 5 [file 41598_2019_53532_MOESM6_ESM.zip › FlySilico/GAM_and_NGAM/ppp_plots/ppp_ATPM_2019_03_15-15_07_47.png]

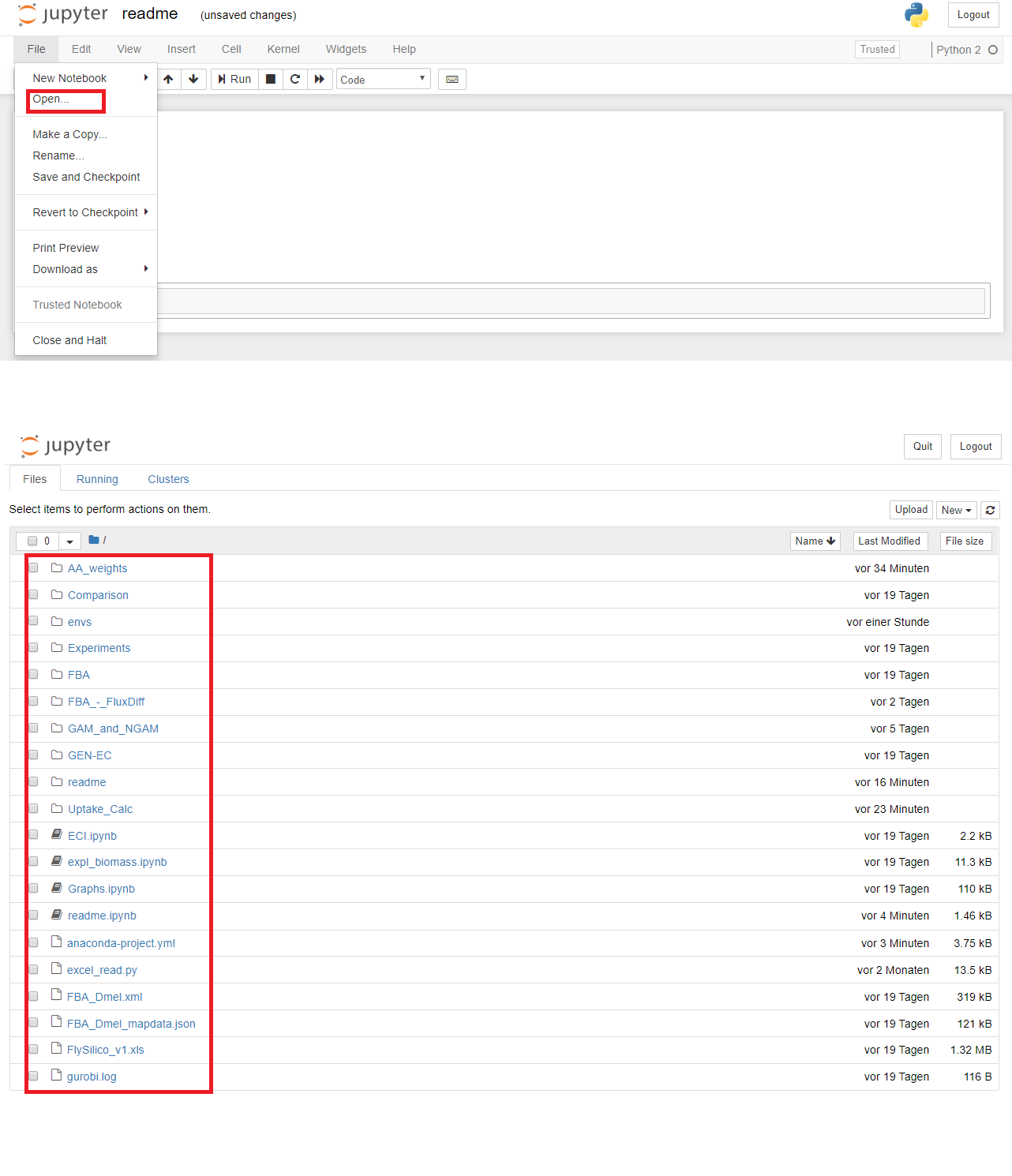

Supplement: Supplementary file 6 — Dataset 5 [file 41598_2019_53532_MOESM6_ESM.zip › FlySilico/readme/readme.png]

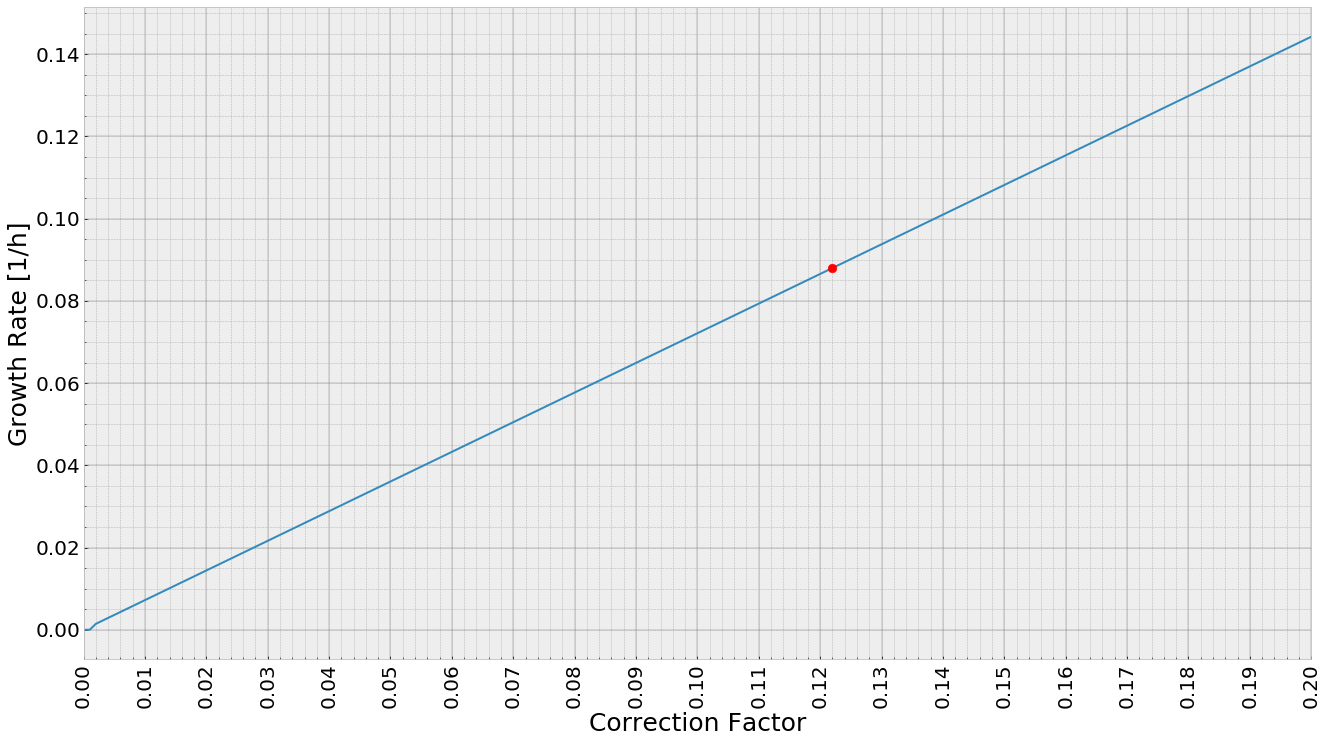

Supplement: Supplementary file 6 — Dataset 5 [file 41598_2019_53532_MOESM6_ESM.zip › FlySilico/Uptake_Calc/correction_factor.png]

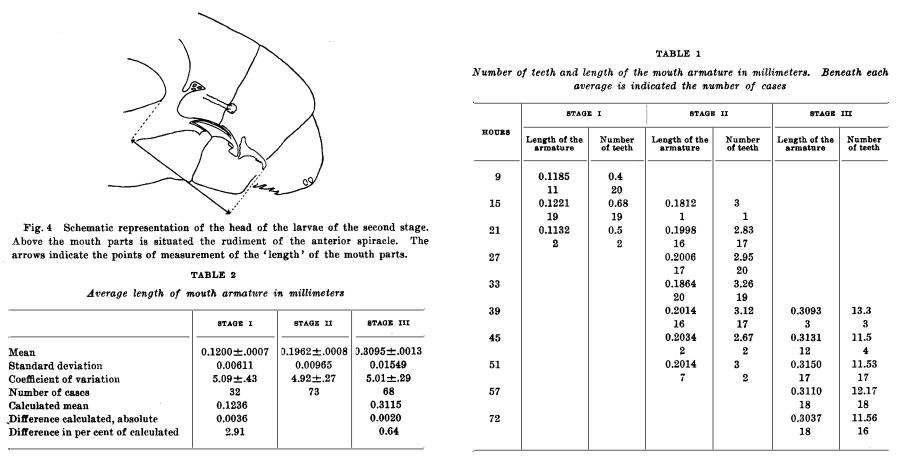

Supplement: Supplementary file 6 — Dataset 5 [file 41598_2019_53532_MOESM6_ESM.zip › FlySilico/Uptake_Calc/mouth.png]
